# Comorbidity and thirty-day hospital readmission odds in chronic obstructive pulmonary disease: a comparison of the Charlson and Elixhauser comorbidity indices

**DOI:** 10.1186/s12913-019-4549-4

**Published:** 2019-10-15

**Authors:** Russell G. Buhr, Nicholas J. Jackson, Gerald F. Kominski, Steven M. Dubinett, Michael K. Ong, Carol M. Mangione

**Affiliations:** 1 Division of Pulmonary and Critical Care Medicine, David Geffen School of Medicine, University of California, 1100 Glendon Ave, Suite 850, Los Angeles, CA 90024 USA; 2 Department of Health Policy and Management, Jonathan and Karin Fielding School of Public Health, University of California, Los Angeles, CA USA; 3 Department of Medicine, Greater Los Angeles Veterans Affairs Healthcare System, Los Angeles, CA USA; 4 Department of Medicine Statistics Core, University of California, Los Angeles, CA USA; 5 Center for Health Policy Research, Jonathan and Karin Fielding School of Public Health, University of California, Los Angeles, CA USA; 6 Division of General Internal Medicine and Health Services Research, David Geffen School of Medicine, University of California, Los Angeles, CA USA

**Keywords:** COPD, Charlson comorbidity index, Elixhauser comorbidity index, Hospital readmission, Nationwide readmissions database

## Abstract

**Background:**

Readmissions following exacerbations of chronic obstructive pulmonary disease (COPD) are prevalent and costly. Multimorbidity is common in COPD and understanding how comorbidity influences readmission risk will enable health systems to manage these complex patients.

**Objectives:**

We compared two commonly used comorbidity indices published by Charlson and Elixhauser regarding their ability to estimate readmission odds in COPD and determine which one provided a superior model.

**Methods:**

We analyzed discharge records for COPD from the Nationwide Readmissions Database spanning 2010 to 2016. Inclusion and readmission criteria from the Hospital Readmissions Reduction Program were utilized. Elixhauser and Charlson Comorbidity Index scores were calculated from published methodology. A mixed-effects logistic regression model with random intercepts for hospital clusters was fit for each comorbidity index, including year, patient-level, and hospital-level covariates to estimate odds of thirty-day readmissions. Sensitivity analyses included testing age inclusion thresholds and model stability across time.

**Results:**

In analysis of 1.6 million COPD discharges, readmission odds increased by 9% for each half standard deviation increase of Charlson Index scores and 13% per half standard deviation increase of Elixhauser Index scores. Model fit was slightly better for the Elixhauser Index using information criteria. Model parameters were stable in our sensitivity analyses.

**Conclusions:**

Both comorbidity indices provide meaningful information in prediction readmission odds in COPD with slightly better model fit in the Elixhauser model. Incorporation of comorbidity information into risk prediction models and hospital discharge planning may be informative to mitigate readmissions.

## Background

The burden of chronic obstructive pulmonary disease (COPD) continues to rise [1, 2], and in the United States, COPD remains the 4th leading cause of death as of 2017 [3]. Exacerbations are common, and economic burdens from related hospitalizations are substantial [4, 5]. In October 2014, the Centers for Medicare and Medicaid Services (CMS) Hospital Readmissions Reduction Program (HRRP) began assessing financial penalties for excessive 30-day hospital readmissions of Medicare patients following COPD hospitalizations [6]. Efforts to understand and reduce readmission risk highly important to health systems. COPD patients have high burdens of various comorbid conditions [7–9], with multiple comorbidities associated with symptom burden, mortality, and hospital utilization [10–13]. COPD patients often meet the threshold of the “multimorbid” patient [14].

The performance of risk adjustment measures in the HRRP remain debated [15], in particular whether risk adjustments adequately control for factors outside the control of a treating hospital. Accurate quantification of comorbidity is crucial for programs that leverage financial penalties to reduce readmissions. Two comorbidity indices are frequently used for research and could potentially adjust for between hospital differences in burden of chronic illness, having previously been shown to be valid for predicting key outcomes of interest [16]. The Charlson Comorbidity Index (CCI) predicts mortality in hospitalized patients [17–19]. Higher CCI scores correlate with mortality, risk of readmission, and lower likelihood of receiving appropriate COPD treatments [20]. The Elixhauser Comorbidity Index (ECI) is an inventory of comorbidities [21], later updated to predict mortality [22, 23] and readmission [24]. Both the Charlson and Elixhauser indices have been associated with readmission outcomes in surgical conditions [25–27], psychiatric conditions [28], and hospitalizations due to other medical conditions and procedures [29–31].

Previous studies show variance between these two indices’ ability to discriminate important outcomes in the COPD population [32]. Understanding the milieu of comorbidity among COPD patients could improve methodology to adjust for readmission risk and enable providers and delivery networks to estimate risk and plan readmission reduction efforts. In order to do this most effectively, an optimal system for quantifying comorbidity and its relationship to readmissions must be identified. Doing so would enable resources within health systems to be directed at those at highest risk of readmission, and also inform policy makers on further improvement in risk stratification methodology within the Hospital Readmissions Reduction Program. Our aim is to compare these two indices’ ability to quantify comorbidity and its contribution to readmission risk in COPD.

## Methods

### Data source

We analyzed discharge records from January 2010 to December 2016 in a pooled, multiple cross-sectional analysis of the Nationwide Readmissions Database (NRD) [33], a nationally representative sample of all-payer discharges from acute care hospitals across multiple states. Because the NRD does not allow for an individual patient to be linked across years, qualifying index discharges were restricted to stays occurring in February through November, as we could not identify whether January stays were actually readmissions from the prior December or follow December index stays into the next January. We restricted to patients who were residents of the state in which they were admitted to avoid loss to follow up when crossing state lines. Sample weights provided with the dataset were applied to calculate national estimates, compensating for under-sampled patient and hospital characteristics [34]. In the analyses that follow, raw numbers indicate the actual observations, while reported percentages and models utilize the sample weights to provide a population estimate.

### Variable construction

We defined an index hospitalization as one where the patient was discharged alive, excluding transfer to other acute care hospitals and discharges against medical advice, occurring at least 30 days since another hospitalization. A COPD stay was defined by principal diagnosis of COPD exacerbation or principal diagnosis of respiratory failure and secondary diagnosis of COPD [35, 36], excluding cases involving lung transplantation. We included all index discharges from the NRD for patients aged ≥40 years with a qualifying COPD diagnosis admitted to a hospital with at least 25 such discharges over the months outlined above for each given year. We defined readmission as return to any hospital for any diagnosis within 30 days of discharge, excluding certain conditions granted exemption from the HRRP (e.g., childbirth, organ transplantation, or chemotherapy). These definitions were constructed to be aligned with published HRRP methodology [35, 36].

Most variables of interest were included in the original dataset; however, we derived several others. The Charlson and Elixhauser comorbidity scores were calculated using ICD codes and Diagnosis Related Groups, using adaptations of published macros [37, 38] to recode individual ICD codes for each diagnosis into the respective comorbidity index categories and calculate weighted scores using the coding schemata outlined by the original comorbidity index publications [24, 39, 40]. We used diagnoses at the time of the index discharge due to limitations of the dataset to identify patients only within each year, precluding a look-back period. We constructed indicators for in-hospital events (e.g.*,* mechanical ventilation) using ICD codes. We estimated the proportion of within-hospital Medicaid patient-days by taking the number of patient-days paid by Medicaid divided by total patient-days each year. We tabulated the number of hospitals visited and admissions within a year to characterize utilization patterns. Hospital volume for all-cause and COPD-specific discharges were tabulated. Additional details on database structure and variable definitions in the online supplemental methods appendix, where a full list of provided and derived covariates can be found (Additional file 1).

### Statistical analysis

Summary statistics were calculated at the patient level, comparing the readmitted and non-readmitted. Continuous variables were compared using Welch’s t-test (i.e., unequal variance), while categorical variables were compared using Chi-squared tests. Readmission rates were aggregated for population estimates by year, quarter, and month. Readmission rates for hospital sub-strata of interest were calculated, with differences across categories estimated by Chi-squared tests. Adjusted readmission odds were estimated using a two-level, mixed-effects logistic regression model with random intercepts assigned at the hospital cluster level using complete case analysis. A threshold of 10% missingness for variables of interest was set a priori to determine the necessity for use of imputation, which was not reached for any variable included in this analysis. We fit separate, parallel models for the Charlson and the Elixhauser indices as primary predictor, with fixed effects for year, patient-discharge- and hospital-level covariates consistent across both models. Comparison of the two models was made using Akaike and Bayesian information criteria, where lower values of the information criterion signify models of better fit [41, 42].

### Sensitivity analysis

We tested the stability of our estimates over time by refitting the model for individual years. We analyzed a liberalized age cutoff to ≥18 years, having initially favored an older age cutoff given the paucity of COPD in younger patients and concern that these observations may represent miscoding. All analyses were performed in Stata version 15.1 (StataCorp, College Station, TX) with weighted estimates reported using patient level survey weights for national representativeness.

## Results

A total of 1,622,983 index COPD admissions (weighted effective sample *N* = 3,743,164) occurred during the seven-year study period, 17.2% of which were readmitted within 30 days of discharge. Patient characteristics are found in Table 1, further stratified by hospital teaching status (Additional file 11) and urban/rural designation (Additional file 12). There were proportionally fewer readmissions among women than men. Readmitted patients were older (68.7 vs 67.9 years). Medicare and Medicaid patients had higher proportions of readmissions than private insurance or self-pay status. Readmitted patients were more frequently discharged to post-acute care or with home health services and had longer lengths of stay (4.16 vs 3.67 days).

**Table 1:** Baseline patient-level characteristics of the aggregated cohort, comparing readmitted to non-readmitted patients in index stays

	Overall *N* = 1,662,983	Not Readmitted *N* = 1,375,099	Readmitted *N* = 287,884	*P*
Sex*, (N) %*				
Male	41.1%	40.8%	42.8%	<.001
Female	58.9%	59.2%	57.2%	
Age*, (N) Mean ± SD*	68.0 ± 11.9	67.9 ± 11.9	68.7 ± 11.7	<.001
Median household income by ZIP code*, (N) %*				
1st Quartile	37.1%	37.0%	37.5%	<.001
2nd Quartile	26.7%	26.8%	26.4%	
3rd Quartile	20.9%	20.9%	20.8%	
4th Quartile	13.9%	13.9%	14.1%	
Missing	1.4%	1.4%	1.4%	
^b^Patient geographic location*, (N) %*				
Central county metro area ≥ 1 M	22.3%	22.0%	23.5%	<.001
Fringe county metro area ≥ 1 M	24.6%	24.4%	25.5%	
County metro area 250,000–999,999 k	20.8%	20.9%	20.4%	
County metro area 50,000–249,999 k	10.3%	10.4%	10.1%	
Micropolitan area	13.0%	13.1%	12.2%	
Non-metro/non-micropolitan (rural)	9.0%	9.1%	8.3%	
^c^Primary Payer*, (N) %*				
Medicare (includes dual-eligible)	70.4%	69.6%	74.3%	<.001
Medicaid	12.0%	11.8%	13.0%	
Private insurance	11.6%	12.3%	8.3%	
Self-pay	3.1%	3.4%	1.9%	
Other, including no-charge	2.9%	3.0%	2.4%	
Number of admissions each patient had over a year*, (N) Mean ± SD*	2.50 ± 1.96	2.13 ± 1.60	4.31 ± 2.50	<.001
Number hospitals where each patient received care over a year*, (N) Mean ± SD*	1.33 ± 0.67	1.31 ± 0.64	1.44 ± 0.75	<.001
Discharge disposition*, (N) %*				
Routine to home	67.5%	69.1%	60.1%	<.001
Transfer to post-acute care	13.1%	12.4%	16.3%	
Other	0.7%	0.7%	0.8%	
Home with home health services	18.7%	17.8%	22.8%	
^a^Length of Stay*, (N) Mean ± SD*	3.75 ± 2.04	3.67 ± 1.96	4.16 ± 2.38	<.001
Care intensity and complications*, (N) %*				
Use of non-invasive ventilation	8.0%	7.7%	9.7%	<.001
Use of mechanical ventilation	4.7%	4.5%	5.7%	<.001
Placement or presents of tracheostomy	0.8%	0.8%	1.2%	<.001
Cardiac arrest	0.2%	0.2%	0.3%	<.001
Performance of cardiopulmonary resuscitation	0.1%	0.1%	0.2%	<.001

Note: Unweighted N’s displayed. Frequencies derived using weighted analysis. ^a^Geometric Mean and SD for log transformed variable presented ^b^N’s 1,373,301 & 287,296; ^c^N’s 1,372,214 & 287,362

**Fig. 1:**
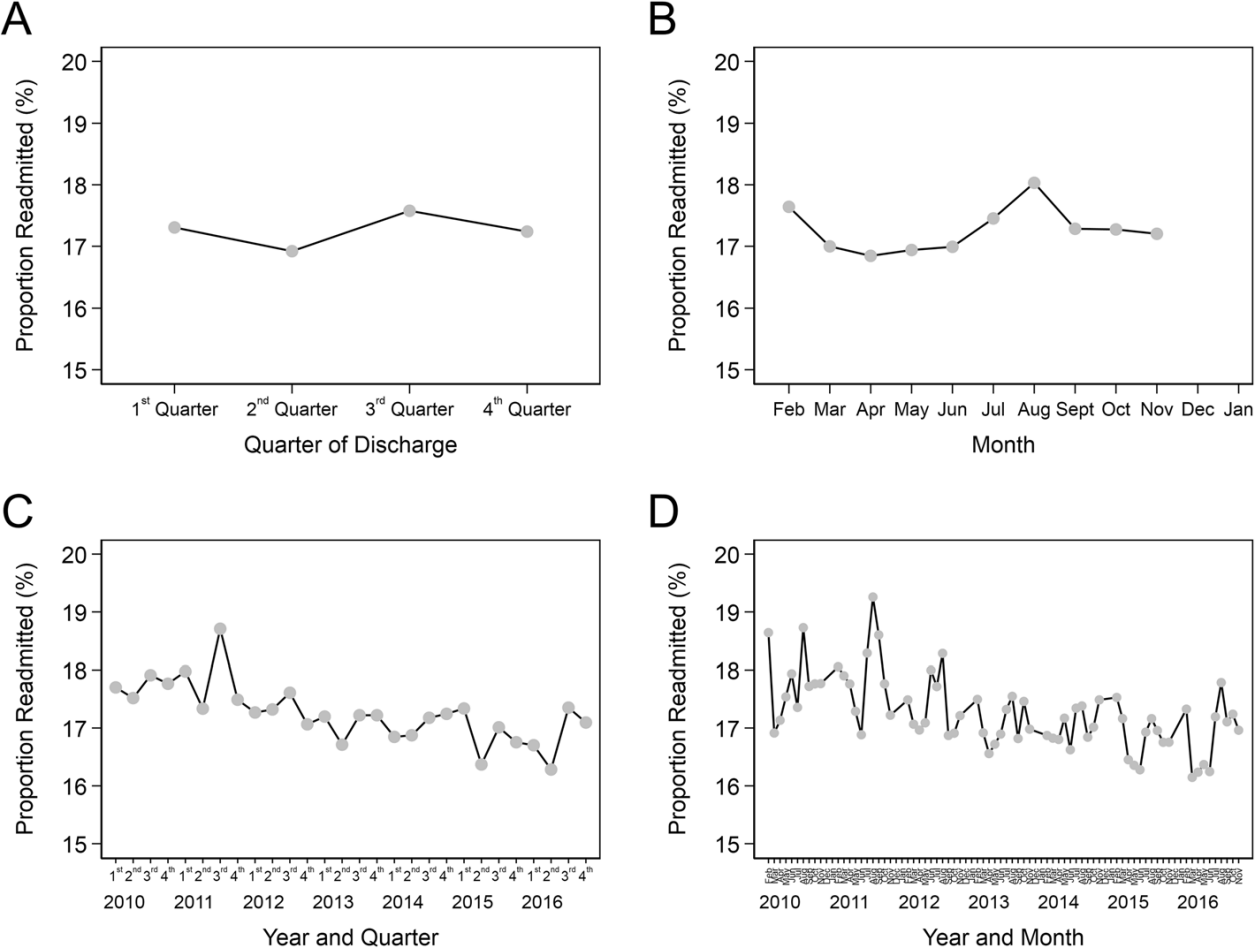
Readmission rates, aggregated within-year by quarter (**a**) and month (**b**), and across-years by quarter (**c**) and month (**d**)

**Fig. 2:**
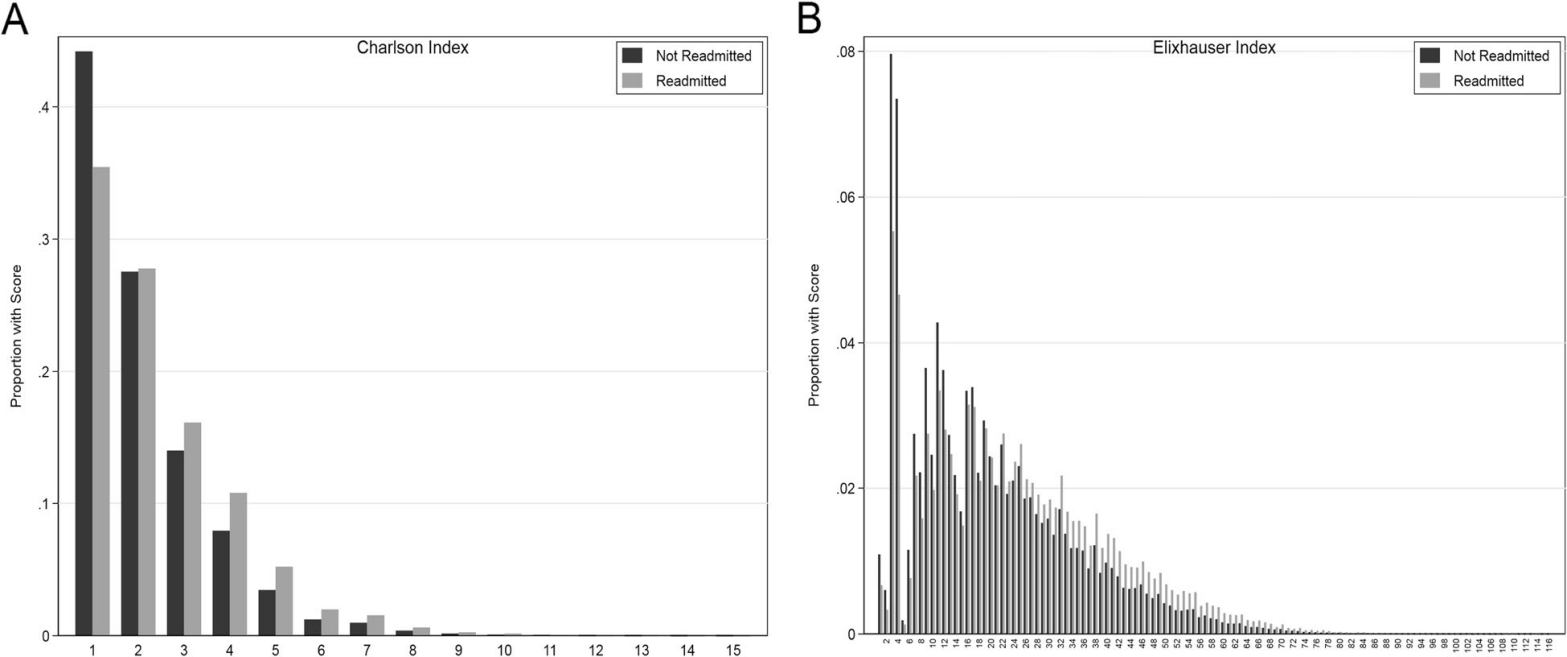
Charlson (**a**) and Elixhauser (**b**) distributions between readmitted and not readmitted patient stays

Hospital characteristics and aggregated sub-cohort readmission rates by hospital type are found in Table 2. In keeping with previous studies, teaching hospitals had higher readmission rates (17.7%) than non-teaching hospitals. For-profit hospitals had a higher readmission rate (17.5%) when compared to governmental (16.8%) and non-profit (17.3%) facilities. Hospitals with higher proportions of Medicaid patients had higher unadjusted readmission rates. There was significant temporal variation in readmission rates both within individual years and across the entire study period, shown in Fig. 1. Distribution of comorbid conditions are shown in Table 3. Readmitted patients had significantly higher mean CCI (2.41 vs. 2.10) and ECI (20.5 vs. 16.3) scores. The distribution of the composite Charlson and Elixhauser scores is shown in Fig. 2. Comorbid conditions were higher across all observed Charlson domains for the readmitted, with the exception of connective tissue diseases. The most pronounced differences were for congestive heart failure (34.8% of readmitted versus 26.1% of non-readmitted) and advanced diabetes (18.1% vs. 13.1%). In Elixhauser categories, readmitted patients had higher proportions of all comorbid conditions with the exception of hypertension, most pronounced for congestive heart failure (34% vs. 25.4%), renal failure (17.2% vs. 12.3%), and iron deficiency anemias (19.9% vs. 14.4%). Average comorbidity scores within for both indices increased over the study period (Additional file 2).

**Table 2:** Baseline characteristics of hospitals included in pooled cohort

	Cohort Proportion	Readmission Rate	*P*
Hospital ownership/control*, (N) %*			
Government, non-federal	16.1%	16.8%	<.001
Private, non-profit	62.9%	17.2%	
Private, for-profit	21.0%	17.4%	
Hospital teaching status*, (N) %*			
Metro, non-teaching	44.2%	17.3%	<.001
Metro, teaching	30.0%	17.6%	
Non-metro, non-teaching	25.8%	15.9%	
Hospital geographic location*, (N) %*			
Large metro area ≥ 1 M	43.7%	17.9%	<.001
Small metro area < 1 M	30.5%	16.8%	
Micropolitan area	15.3%	16.0%	
Non-metro/non-micropolitan (rural)	10.5%	15.5%	
Hospital bed size*, (N) %*			
Small	26.6%	16.5%	<.001
Medium	32.3%	17.1%	
Large	41.1%	17.4%	
Hospital total all-cause annual discharges*, (N) Mean ± SD*	6296 ± 6425		
Quartiles of Hospital total all-cause annual discharges*, (N) %*			
1st Quartile (≤ 8971)	59.1%	16.4%	<.001
2nd Quartile (8972 – 15,406)	20.9%	17.5%	
3rd Quartile (15,407 – 24,534)	12.9%	17.7%	
4th Quartile (≥24,535)	7.1%	18.0%	
COPD Discharges*, (N) Mean ± SD*	161 ± 133		
COPD Discharge Quartiles			
1st Quartile (≤ 122)	48.5%	15.8%	<.001
2nd Quartile (123–205)	24.1%	17.0%	
3rd Quartile (206–322)	17.0%	17.6%	
4th Quartile (≥ 323)	10.4%	18.0%	
Proportion of Medicaid patient days*, (N) Mean ± SD*	0.171 ± 0.112		
Medicaid Proportion Quartiles*, (N) %*			
1st Quartile (≤ 10.6%)	31.5%	16.9%	<.001
2nd Quartile (10.6–16.1%)	25.1%	17.2%	
3rd Quartile (16.1–23.9%)	22.9%	17.3%	
4th Quartile (≥ 23.9%)	20.5%	17.6%	

Note: Unweighted frequencies displayed for cohort proportions. Weighted frequencies for Sub-Strata readmission rates presented. *P* values are for between hospital characteristic differences in readmission rates

**Table 3:** Baseline comorbidity characteristics of the aggregated cohort, comparing readmitted to non-readmitted patients in index stays for the Charlson and Elixhauser Indices

	Overall (1,622,983)	Not Readmitted (1,375,099)	Readmitted (287,884)	*P*
Charlson Index Composite	2.16 ± 1.43	2.10 ± 1.39	2.41 ± 1.57	<.001
Charlson Index Grouping				
Index Score = 1	42.7%	44.2%	35.5%	<.001
Index Score = 2	27.6%	27.5%	27.8%	
Index Score ≥ 3	29.7%	28.3%	36.8%	
Charlson Component Comorbidities				
*Neurologic/Psychiatric*				
Cerebrovascular disease	3.1%	3.0%	3.5%	<.001
Dementia	1.8%	1.8%	1.9%	0.041
Hemiplegia/paraplegia	0.3%	0.3%	0.3%	<.001
*Cardiovascular*				
Congestive heart failure	27.6%	26.1%	34.8%	<.001
Peripheral vascular disease	5.6%	5.4%	6.2%	<.001
Myocardial infarction	7.8%	7.6%	8.9%	<.001
*Respiratory*				
Chronic pulmonary disease	100.0%	100.0%	100.0%	–
*Gastrointestinal*				
Peptic ulcer disease	0.8%	0.8%	0.9%	<.001
Mild liver disease	2.2%	2.1%	2.4%	<.001
Moderate or severe liver disease	0.2%	0.1%	0.2%	<.001
*Renal/Electrolyte Disorders*				
Moderate or severe renal disease	14.0%	13.1%	18.1%	<.001
*Infectious Disease*				
HIV/AIDS	0.2%	0.2%	0.3%	<.001
*Hematologic/Oncology*				
Malignancy (any type)	3.4%	3.2%	4.4%	<.001
Metastatic solid tumor	1.1%	1.0%	1.7%	<.001
*Rheumatologic and Musculoskeletal*				
Connective tissue disease	2.2%	2.2%	2.2%	0.131
*Endocrine*				
Diabetes mellitus	16.5%	16.4%	17.0%	<.001
Diabetes mellitus w/end-organ damage	2.1%	2.0%	2.6%	<.001
Elixhauser Index Composite	17.0 ± 15.0	16.3 ± 14.7	20.5 ± 16.0	<.001
Elixhauser Comorbidity Domain Count	3.99 ± 1.84	3.92 ± 1.81	4.37 ± 1.91	<.001
Elixhauser Component Comorbidities				
*Neurologic/Psychiatric*				
Paralysis	1.3%	1.2%	1.5%	<.001
Other neurologic disorders	8.6%	8.3%	9.8%	<.001
Alcohol abuse	4.5%	4.5%	4.7%	<.001
Drug abuse	3.6%	3.5%	4.2%	<.001
Psychoses	6.3%	6.1%	7.6%	<.001
Depression	16.9%	16.7%	17.8%	<.001
*Cardiovascular*				
Congestive heart failure	26.9%	25.4%	34.0%	<.001
Peripheral vascular disease	7.9%	7.7%	9.2%	<.001
Valvular Heart Disease	6.5%	6.2%	7.5%	<.001
Hypertension	54.1%	54.5%	52.2%	<.001
*Respiratory*				
Chronic pulmonary disease	100.0%	100.0%	100.0%	–
Pulmonary circulation disorders	7.9%	7.6%	9.8%	<.001
*Gastrointestinal*				
Peptic ulcer disease	0.1%	0.1%	0.2%	<.001
Liver disease	2.4%	2.3%	2.8%	<.001
*Renal/Electrolyte Disorders*				
Renal Failure	13.1%	12.3%	17.2%	<.001
Fluid and electrolyte disorders	28.0%	27.4%	30.9%	<.001
*Infectious Disease*				
HIV/AIDS	0.2%	0.2%	0.3%	<.001
*Hematologic/Oncology*				
Solid tumor without metastasis	3.4%	3.1%	4.6%	<.001
Metastatic cancer	1.1%	1.0%	1.7%	<.001
Lymphoma	0.5%	0.5%	0.6%	<.001
Coagulopathy	3.2%	3.1%	3.8%	<.001
Blood loss anemia	0.4%	0.3%	0.5%	<.001
Deficiency anemia	15.4%	14.4%	19.9%	<.001
*Rheumatologic and Musculoskeletal*				
Rheumatoid arthritis and collagen vascular disorders	3.3%	3.3%	3.5%	<.001
*Endocrine*				
Diabetes mellitus (uncomplicated)	26.3%	25.8%	28.4%	<.001
Diabetes mellitus (complicated)	6.0%	5.7%	7.3%	<.001
Hypothyroidism	13.7%	13.6%	14.0%	<.001
Obesity	19.1%	19.0%	19.6%	<.001
Weight loss	4.6%	4.5%	5.4%	<.001

Note: Unweighted N’s displayed. Frequencies derived using weighted analysis

Multi-level logistic regression models were fitted separately for the Charlson Index and the Elixhauser Index and compared (Table 4). To standardize comparisons between the models, the comorbidity indices were scaled by their distributions, such that an odds ratio was calculated for a change of one-half standard deviation (SD) in score. In our adjusted models, a 1/2 SD change in the Charlson score (~ 1.5 points) was associated with a 9% increase in readmission odds while a 1/2 SD change in the Elixhauser score (~ 7.5 points) portended a 13% increase in readmission odds. The unadjusted (Model 1), patient-adjusted (Model 2), and patient- and hospital-adjusted (Model 3) estimates in their original scaling are found in Additional file 3 (CCI) and Additional file 4 (ECI).

**Table 4:** Multilevel logistic regression model for Charlson (left) and Elixhauser (right) Indices, adjusted for patient and hospital factors with random intercepts for hospital clusters

Model Info	Charlson Index		Elixhauser Index	
N	1,658,372		1,658,372	
LL	-1,683,418.10		-1,677,856.30	
df	41		41	
AIC	3,366,918.30		3,355,794.50	
BIC	3,367,423.50		3,356,299.80	
Predictors	OR (95% CI)	*P*	OR (95% CI)	*P*
Comorbidity Index *(per ½ SD)*	1.09 (1.09, 1.09)	<.001	1.13 (1.12, 1.13)	<.001
Year *(ref = 2010)*				
*2011*	1.00 (0.97, 1.02)	0.673	0.99 (0.97, 1.01)	0.484
*2012*	0.96 (0.94, 0.98)	<.001	0.95 (0.93, 0.97)	<.001
*2013*	0.93 (0.91, 0.95)	<.001	0.91 (0.89, 0.93)	<.001
*2014*	0.92 (0.90, 0.94)	<.001	0.89 (0.87, 0.91)	<.001
*2015*	0.88 (0.86, 0.90)	<.001	0.86 (0.84, 0.88)	<.001
*2016*	0.87 (0.85, 0.89)	<.001	0.85 (0.83, 0.87)	<.001
Quarter *(ref = 1st)*				
*2nd Quarter*	0.97 (0.96, 0.98)	<.001	0.97 (0.95, 0.98)	<.001
*3rd Quarter*	1.01 (1.00, 1.02)	0.167	1.00 (0.99, 1.02)	0.778
*4th Quarter*	0.99 (0.98, 1.01)	0.277	0.99 (0.97, 1.00)	0.086
Sex *(ref = male)*				
*Female*	0.92 (0.91, 0.93)	<.001	0.92 (0.91, 0.92)	<.001
Age *(per 10 year)*	0.97 (0.97, 0.98)	<.001	0.98 (0.98, 0.99)	<.001
Income Quartile *(ref = 1st)*				
*2nd Quartile*	0.98 (0.97, 0.99)	0.003	0.98 (0.97, 1.00)	0.009
*3rd Quartile*	0.97 (0.95, 0.98)	<.001	0.97 (0.96, 0.99)	<.001
*4th Quartile*	0.94 (0.93, 0.96)	<.001	0.95 (0.93, 0.97)	<.001
*Missing*	0.97 (0.93, 1.00)	0.083	0.97 (0.93, 1.01)	0.136
Payer *(ref = Medicare)*				
*Medicaid*	1.08 (1.06, 1.10)	<.001	1.08 (1.06, 1.09)	<.001
*Private*	0.69 (0.68, 0.71)	<.001	0.71 (0.70, 0.73)	<.001
*Self-Pay*	0.62 (0.60, 0.64)	<.001	0.63 (0.61, 0.65)	<.001
*Other/No Charge*	0.79 (0.77, 0.82)	<.001	0.80 (0.77, 0.82)	<.001
Disposition *(ref = Routine to home)*				
*Post-acute care*	1.30 (1.28, 1.32)	<.001	1.21 (1.19, 1.23)	<.001
*Home Health*	1.35 (1.33, 1.37)	<.001	1.30 (1.28, 1.32)	<.001
*Other*	1.13 (1.06, 1.20)	<.001	1.07 (1.00, 1.14)	0.038
Length of Stay *(per day)*	1.02 (1.02, 1.02)	<.001	1.01 (1.01, 1.01)	<.001
Care intensity *(ref = No)*				
*Non-invasive ventilation*	1.16 (1.14, 1.18)	<.001	1.08 (1.06, 1.10)	<.001
*Mechanical ventilation*	0.95 (0.92, 0.97)	<.001	0.82 (0.79, 0.84)	<.001
*Tracheostomy*	1.02 (0.97, 1.09)	0.425	1.04 (0.99, 1.11)	0.133
*Cardiac arrest*	0.82 (0.74, 0.91)	<.001	0.81 (0.74, 0.90)	<.001
*CPR*	1.08 (0.95, 1.24)	0.242	1.09 (0.95, 1.24)	0.21
Hospital ownership *(ref = government)*				
*Private, non-profit*	0.99 (0.98, 1.01)	0.437	0.98 (0.96, 1.00)	0.022
*Private, for-profit*	1.04 (1.01, 1.06)	<.001	1.03 (1.01, 1.06)	0.002
Hospital teaching status *(ref = Non-teaching)*				
*Teaching Hospital*	1.01 (0.99, 1.02)	0.423	1.00 (0.98, 1.01)	0.902
Hospital location *(ref = Large metro area)*				
*Small metro area*	0.94 (0.92, 0.95)	<.001	0.93 (0.92, 0.95)	<.001
*Micropolitan area*	0.89 (0.87, 0.91)	<.001	0.90 (0.88, 0.92)	<.001
*Rural*	0.87 (0.84, 0.90)	<.001	0.89 (0.86, 0.92)	<.001
Hospital Bed Size *(ref = Small)*				
*Medium*	1.01 (0.99, 1.03)	0.174	1.01 (0.99, 1.03)	0.389
*Large*	1.02 (1.00, 1.04)	0.04	1.01 (0.99, 1.03)	0.407
Annual Discharge (per 10 k)	1.01 (1.00, 1.02)	0.13	1.01 (1.00, 1.02)	0.012
Proportion of Medicaid patient days per 10%	1.00 (1.00, 1.01)	0.531	1.00 (0.99, 1.01)	0.872

Women had lower odds than men and readmission odds decreased with increases in age. In both models, Medicaid patients had higher readmission odds and the privately insured had lower odds compared to Medicare. Hospital proportion of Medicaid patient-days did not correlate with readmission odds. Compared to routine discharges home, those with transfers to post-acute care and home with home health services had significantly higher readmission odds. Each day increase in length of stay portended slightly higher readmission odds. Smaller but significant effects were observed for hospital location, while hospital teaching status, volume of discharges. The effect sizes for covariates were similar for both comorbidity models. To compare the fit of the two models, we employed the Akaike (AIC) and Bayesian (BIC) information criteria, which deal with the balance between goodness-of-fit and parsimony of any given model [41, 42]. In our analysis, the Elixhauser model had a lower AIC (3,355,795 vs. 3,366,918) and BIC (3,356,300 vs, 3,367,434) than the Charlson, demonstrating better fit.

In the sensitivity analyses, the odds ratios for comorbidity indices did not vary significantly across time (Additional file 5 and Additional file 6). When we evaluated whether our original age restriction to patients ≥40 years old had any significant influence on the models, we found no substantial changes in our estimates by expanding to all adults ≥18 years old (Additional file 7 for CCI and Additional file 8 for ECI). We also evaluated whether the presence of the comorbidity index substantially changed the estimates of the covariates by fitting an additional model without the comorbidity indices (Additional file 9) which showed small changes in effect sizes, but no sign changes between the reduced and original models, with better fit in our original model (Additional file 10). Of notable difference, the effects of discharge disposition and care intensity diminished, likely demonstrating some collinearity between these factors and comorbidity.

## Discussion

In this large, all-payer, population study of those admitted COPD exacerbations, we examined the contributions of comorbidity and key patient and hospital factors to risk for 30-day rehospitalization. Escalating degrees of comorbidity were associated with significantly higher odds of 30-day readmission even after controlling for other patient and hospital factors in both the Charlson and Elixhauser models. This is in line with previously published work in the Veterans Affairs population showing that higher Charlson scores were associated with higher risk of readmissions [20], and a new finding for use of the Elixhauser model in COPD. While the comparison of comorbidity indices has been previously published with regard to predicting COPD exacerbations and hospitalizations [32], our findings showing the comparison of two widely-used candidate comorbidity indices to predict readmissions is novel. In this comparison, the Elixhauser Comorbidity Index performed slightly better, with the inclusion of more comorbidity parameters giving a better model fit.

While previous studies of Medicare patients for other HRRP conditions have not shown socioeconomic status to be consistently correlated with readmission outcomes [43, 44], our study showed that patients who lived in higher income neighborhoods had progressively lower adjusted readmission odds. Furthermore, while other studies in these populations have raised concern about higher readmission burdens in hospitals serving lower-income patients [45–48], our study did not show a significant correlation between adjusted readmission odds and proportion of hospital Medicaid patient-days. Our methodology did not allow for exact approximation of Disproportionate Share Hospital estimates due to lack of information on supplemental security income [49], and using Medicaid as a proxy may underrepresent the burdens on safety-net hospitals. These findings may be at odds with the arguments for need for additional adjustments for socioeconomic factors, but given the proxies used to measure income status in this study, it is difficult to draw a definitive conclusion from these data.

In sensitivity analyses, we found that including comorbidity scores improved upon models simply using patient demographic and hospital characteristics. This is unsurprising, given the rich detail that comorbidity information adds. The fact that comorbidity scores increased with time may reflect coding practice changes, as health systems include additional comorbid conditions in their discharge diagnoses to increase the severity reflected in coding schema for Diagnosis Related Grouping, or to mitigate readmission penalties by shoring up risk categorization [50]. It is possible that transition to electronic health records under the Affordable Care Act’s Meaningful Use provisions let to more precise coding of diagnoses, though our data source does not allow for this delineation. In addition, more diagnoses (25 per record 2010–13, 30 in 2014, and 35 in 2015–16) are included in the later years of the dataset, which has been associated with up-coding of severity in Medicare analyses [51]. Regardless, our estimates for readmission odds were stable across time despite the decreases in readmission rates and the increases in coded comorbidity and transition from ICD-9 to ICD-10.

### Limitations and residual confounding

Inconsistent coding of comorbid conditions at the time of hospital discharge may hinder our ability to truly model the breadth of comorbidity in our study. We were also limited by the structure of the database, using pooled cross-sectional data instead of a true longitudinal sample, further limited by the database’s inability to identify the same patient or hospital across years. As such, there were likely some cases where a patient was measured more than once but not identified as such. While some degree of auto-correlation is possible from our approach, the large sample size was felt to adequately compensate for this. The same is true of repeated visits from the same patient within a year introducing additional correlation between readmissions resulting in potentially overly narrow confidence intervals. The NRD as a data source contains some inherent limitations, including the absence of a race variable and the coding of income by medians at ZIP-centers rather than being patient-reported [52]. Inability to track a patient across state lines may lead to under-reporting of readmissions. Furthermore, not all states are included in the database, however provided sampling weights were used to compensate for otherwise underrepresented patient and hospital types [34].

Within these limitations, however, our approach fills in important gaps in the currently published literature. By including an all-payer sample, we are able to better understand the patient milieu beyond the Medicare population, where most previous studies have been done. The sample is nationally representative and covers all community hospital discharges within the study period across a wide range of states. Use of such a broad patient population enables insights not previously afforded by individual health system or payer populations.

## Conclusion

In a large, national, all-payer sample of COPD hospitalizations, comorbidities are frequent and play a substantial role in the 30-day readmission risk. Between two available comorbidity scoring systems, the Elixhauser Comorbidity Index provides better model fit when compared to the Charlson Comorbidity Index and should be favored for future analyses of this type. Using comorbidity in risk adjustment tools may provide policy makers with additional insight into how best to correct for the multimorbid patient when assessing penalties. In addition, health systems seeking to improve their delivery methods could use such a scoring system to better understand their own distribution of comorbidities in order to develop programs tailored to their individual patient populations. Further study of the differential influence of these comorbid conditions on outcomes and the mitigating effects of care delivery by integrated practice units to address multimorbidity is warranted.

## Supplementary information

**Additional file 1.** Supplemental Methods Appendix.

**Additional file 2:** **Figure S1.** Trend of mean Charlson and Elixhauser Index scores over time.

**Additional file 3: Table S1.** Multilevel Logistic Regression models of Readmission using Charlson Index using Hospital Level random intercept.

**Additional file 4: Table S2.** Multilevel Logistic Regression models of Readmission using Elixhauser Index using Hospital Level random intercept.

**Additional file 5: Table S3.** Charlson and Elixhauser Indices (with 95% CI) over time per 1/2 standard deviation increase.

**Additional file 6: Figure S2.** Changes in Charlson and Elixhauser Indices (with 95% CI) overtime per 1/2 standard deviation increase.

**Additional file 7: Table S4.** Multilevel Logistic Regression models of Readmission using Charlson Index using Hospital Level random intercept (age 18 and older).

**Additional file 8: Table S5.** Multilevel Logistic Regression models of Readmission using Elixhauser Index using Hospital Level random intercept (age 18 and older).

**Additional file 9: Table S6.** Multilevel Logistic Regression models of Readmission using only the covariates as predictor with Hospital Level random intercept.

**Additional file 10: Table S7.** Comparisons between covariate-only and comorbidity index models.

**Additional file 11: Table S8.** Pooled cohort characteristics of patients, stratified by hospital teaching status.

**Additional file 12: Table S9.** Pooled cohort characteristics of patients, stratified by urban/rural hospital location designation.

## Data Availability

The datasets generated and/or analyzed during the current study are available in the Agency for Healthcare Research and Quality’s Healthcare Utilization Project repository, available online at https://www.hcup-us.ahrq.gov/nrdoverview.jsp. A full list of partner organizations providing data for the Nationwide Readmission Database can be found at https://www.hcup-us.ahrq.gov/db/hcupdatapartners.jsp.

## Abbreviations

AHRQ: Agency for Healthcare Research and Quality
CCI: Charlson comorbidity index
CMS: Centers for Medicare and Medicaid Services
COPD: chronic obstructive pulmonary disease
ECI: Elixhauser comorbidity index
HCUP: Healthcare Cost and Utilization Project
HRRP: Hospital Readmissions Reduction Program
ICD: International Classification of Diseases
NRD: Nationwide Readmissions Database
SD: Standard deviation

## References

[1] Ford ES, Croft JB, Mannino DM, Wheaton AG, Zhang X, Giles WH (2013). COPD surveillance--United States, 1999-2011. Chest.

[2] Mannino DM, Homa DM, Akinbami LJ, Ford ES, Redd SC (2002). Chronic obstructive pulmonary disease surveillance--United States, 1971-2000. MMWR Surveill Summ.

[3] Murphy SL, Xu JQ, Kochanek KD, Arias E (2018). Mortality in the United States, 2017. Centers for Disease Control and Prevention.

[4] Guarascio AJ, Ray SM, Finch CK, Self TH (2013). The clinical and economic burden of chronic obstructive pulmonary disease in the USA. Clinicoecon Outcomes Res.

[5] Press VG, Konetzka RT, White SR (2018). Insights about the economic impact of chronic obstructive pulmonary disease readmissions post implementation of the hospital readmission reduction program. Curr Opin Pulm Med.

[6] Centers for Medicare & Medicaid Services. Readmissions Reduction Program 2017. updated 30 November 2017. Available from: https://www.cms.gov/Medicare/Medicare-Fee-for-Service-Payment/AcuteInpatientPPS/Readmissions-Reduction-Program.html.

[7] Kahnert K, Alter P, Young D, Lucke T, Heinrich J, Huber RM (2018). The revised GOLD 2017 COPD categorization in relation to comorbidities. Respir Med.

[8] Global Initiative for Chronic Obstructive Lung Disease (2019). Global Strategy for the Prevention, Diagnosis, and Management of COPD Fontana, WI.

[9] Zewari S, Hadi L, van den Elshout F, Dekhuijzen R, Heijdra Y, Vos P (2018). Obesity in COPD: comorbidities with practical consequences?. COPD.

[10] Gudmundsson G, Gislason T, Lindberg E, Hallin R, Ulrik CS, Brondum E (2006). Mortality in COPD patients discharged from hospital: the role of treatment and co-morbidity. Respir Res.

[11] Roberts CM, Stone RA, Lowe D, Pursey NA, Buckingham RJ (2011). Co-morbidities and 90-day outcomes in hospitalized COPD exacerbations. COPD.

[12] Prudente R, Franco EAT, Mesquita CB, Ferrari R, de Godoy I, Tanni SE (2018). Predictors of mortality in patients with COPD after 9 years. Int J Chron Obstruct Pulmon Dis.

[13] Jeong SH, Lee H, Carriere KC, Shin SH, Moon SM, Jeong BH (2016). Comorbidity as a contributor to frequent severe acute exacerbation in COPD patients. Int J Chron Obstruct Pulmon Dis.

[14] Westney G, Foreman MG, Xu J, Henriques King M, Flenaugh E, Rust G (2017). Impact of comorbidities among Medicaid enrollees with chronic obstructive pulmonary disease, United States, 2009. Prev Chronic Dis.

[15] Thompson MP, Kaplan CM, Cao Y, Bazzoli GJ, Waters TM (2016). Reliability of 30-day readmission measures used in the hospital readmission reduction program. Health Serv Res.

[16] Austin SR, Wong Y-N, Uzzo RG, Beck JR, Egleston BL (2015). Why summary comorbidity measures such as the Charlson comorbidity index and Elixhauser score work. Med Care.

[17] Charlson ME, Pompei P, Ales KL, MacKenzie CR (1987). A new method of classifying prognostic comorbidity in longitudinal studies: development and validation. J Chronic Dis.

[18] Charlson M, Szatrowski TP, Peterson J, Gold J (1994). Validation of a combined comorbidity index. J Clin Epidemiol.

[19] Deyo RA, Cherkin DC, Ciol MA (1992). Adapting a clinical comorbidity index for use with ICD-9-CM administrative databases. J Clin Epidemiol.

[20] Spece LJ, Epler EM, Donovan LM, Griffith MF, Collins MP, Feemster LC (2018). Role of comorbidities in treatment and outcomes after chronic obstructive pulmonary disease exacerbations. Ann Am Thor Soc.

[21] Elixhauser A, Steiner C, Harris DR, Coffey RM (1998). Comorbidity measures for use with administrative data. Med Care.

[22] van Walraven C, Austin PC, Jennings A, Quan H, Forster AJ (2009). A modification of the Elixhauser comorbidity measures into a point system for hospital death using administrative data. Med Care.

[23] Thompson NR, Fan Y, Dalton JE, Jehi L, Rosenbaum BP, Vadera S (2015). A new Elixhauser-based comorbidity summary measure to predict in-hospital mortality. Med Care.

[24] Moore BJ, White S, Washington R, Coenen N, Elixhauser A (2017). Identifying increased risk of readmission and in-hospital mortality using hospital administrative data: the AHRQ Elixhauser comorbidity index. Med Care.

[25] McFerrin C, Raza SJ, May A, Davaro F, Siddiqui S, Hamilton Z. Charlson comorbidity score is associated with readmission to the index operative hospital after radical cystectomy and correlates with 90-day mortality risk. Int Urol Nephrol. 2019. 10.1007/s11255-019-02247-6 31346955

[26] Voskuijl T, Hageman M, Ring D (2014). Higher Charlson comorbidity index scores are associated with readmission after orthopaedic surgery. Clin Orthop Relat Res.

[27] Mehta HB, Dimou F, Adhikari D, Tamirisa NP, Sieloff E, Williams TP (2016). Comparison of comorbidity scores in predicting surgical outcomes. Med Care.

[28] Sprah L, Dernovsek MZ, Wahlbeck K, Haaramo P (2017). Psychiatric readmissions and their association with physical comorbidity: a systematic literature review. BMC Psychiatry.

[29] Arora S, Lahewala S, Hassan Virk HU, Setareh-Shenas S, Patel P, Kumar V (2017). Etiologies, trends, and predictors of 30-day readmissions in patients with diastolic heart failure. Am J Cardiol.

[30] Gadre SK, Shah M, Mireles-Cabodevila E, Patel B, Duggal A (2019). Epidemiology and predictors of 30-day readmission in patients with sepsis. Chest.

[31] Dhakal B, Giri S, Levin A, Rein L, Fenske TS, Chhabra S (2019). Factors associated with unplanned 30-day readmissions after hematopoietic cell transplantation among US hospitals. JAMA Netw Open.

[32] Austin PC, Stanbrook MB, Anderson GM, Newman A, Gershon AS (2012). Comparative ability of comorbidity classification methods for administrative data to predict outcomes in patients with chronic obstructive pulmonary disease. Ann Epidemiol.

[33] HCUP Nationwide readmissions database (NRD). Agency for Healthcare Research and Quality. 2010-2016. Available from: https://www.hcup-us.ahrq.gov/nrdoverview.jsp.

[34] Healthcare Cost and Utilization Project. Introduction to the HCUP Nationwide Readmissions Database (NRD) 2010-2016, Agency for Healthcare Research and Quality. Rockville; 2018. updated August 2018. Available from: https://www.hcup-us.ahrq.gov/db/nation/nrd/Introduction_NRD_2010-2016.jsp

[35] Yale New Haven Health Services Corporation/Center for Outcomes Research & Evaluation (2016). 2016 condition-specific measures updates and specifications report hospital-level 30-day risk-standardized readmission measures.

[36] Yale New Haven Health Services Corporation/Center for Outcomes Research & Evaluation (2017). 2017 condition-specific measures updates and specifications report hospital-level 30-day risk-standardized readmission measures.

[37] Stagg V (2006). CHARLSON: Stata module to calculate Charlson index of comorbidity. Boston College Department of Economics: Statistical Software Components.

[38] Stagg V (2015). ELIXHAUSER: Stata module to calculate Elixhauser index of comorbidity. Boston College Department of Economics: Statistical Software Components.

[39] Sundararajan V, Henderson T, Perry C, Muggivan A, Quan H, Ghali WA (2004). New ICD-10 version of the Charlson comorbidity index predicted in-hospital mortality. J Clin Epidemiol.

[40] Healthcare Cost and Utilization Project (2017). HCUP Elixhauser Comorbidity Software. Rockville: Agency for Healthcare Research and Quality.

[41] Bozdogan H (1987). Model selection and Akaike's information criterion (AIC): the general theory and its analytical extensions. Psychometrika.

[42] Vrieze SI (2012). Model selection and psychological theory: a discussion of the differences between the Akaike information criterion (AIC) and the Bayesian information criterion (BIC). Psychol Methods.

[43] Bernheim SM, Parzynski CS, Horwitz L, Lin Z, Araas MJ, Ross JS (2016). Accounting for Patients' socioeconomic status does not change hospital readmission rates. Health Aff (Millwood).

[44] Nagasako EM, Reidhead M, Waterman B, Claiborne DW (2014). Adding socioeconomic data to hospital readmissions calculations May produce more useful results. Health Aff (Millwood).

[45] Fuller RL, Atkinson G, Hughes JS (2015). Indications of biased risk adjustment in the hospital readmission reduction program. J Ambul Care Manage.

[46] Sjoding MW, Cooke CR (2014). Readmission penalties for chronic obstructive pulmonary disease will further stress hospitals caring for vulnerable patient populations. Amer J Resp Crit Care Med.

[47] Prieto-Centurion V, Gussin HA, Rolle AJ, Krishnan JA (2013). Chronic obstructive pulmonary disease readmissions at minority-serving institutions. Ann Am Thor Soc..

[48] Caracciolo C, Parker D, Marshall E, Brown J (2017). Excess readmission vs excess penalties: maximum readmission penalties as a function of socioeconomics and geography. J Hosp Med.

[49] Centers for Medicare & Medicaid Services (2018). Disproportionate Share Hospital (DSH).

[50] Steinwald B, Dummit LA (1989). Hospital case-mix change: sicker patients or DRG creep?. Health Aff (Millwood).

[51] Tsugawa Y, Figueroa JF, Papanicolas I, Orav EJ, Jha AK. Assessment of Strategies for Managing Expansion of Diagnosis Coding Using Risk-Adjustment Methods for Medicare. JAMA Int Med. 2019;179(9):1287-90. 10.1001/jamainternmed.2019.1005 PMC6596333 31242282

[52] Healthcare Cost and Utilization Project (2018). NRD description of data elements Rockville, MD: Agency for Healthcare Research and Quality.

