# Supplementary material for: Comorbidity and thirty-day hospital readmission odds in chronic obstructive pulmonary disease: a comparison of the Charlson and Elixhauser comorbidity indices
Source: BMC Health Serv Res. 2019 Oct 15;19:701. doi: 10.1186/s12913-019-4549-4 (PMC6794890; doi:10.1186/s12913-019-4549-4)
Supplement: Supplementary file 1 — Additional file 1. Supplemental Methods Appendix. [file 12913_2019_4549_MOESM1_ESM.docx]

**Comorbidity and thirty-day hospital readmission odds in chronic obstructive pulmonary disease: a comparison of the Charlson and Elixhauser Comorbidity Indices**

# Online Supplemental Appendix

## Detailed Methodology

The Nationwide Readmission Database (NRD) consists of yearly cross-sectional samples abstracted from the State Inpatient Databases. This database fills a gap in currently available knowledge about rehospitalizations, as there has not previously been an all-payer, nationally representative sample from which to estimate hospital readmissions. The Agency for Healthcare Research and Quality (AHRQ) maintains this database, which is compiled from discharge data from the State Inpatient Databases of multiple states (1). For the purposes of this analysis, the databases from 2010-2016 are being included, which represent all currently available data at the time of this submission.

### Data Source and Construction

The NRD is aggregated from State Inpatient Databases, maintained by the AHRQ. Sixteen states appear in all 7 years included in the analyses, with a maximum number of 27 states in the 2015 and 2016 datasets and a minimum of 18 states in 2010-2012, with a summary of the states included provided below in Supplemental Table 1 (2). Individual observations in the dataset represent hospital discharge records, summarizing key administrative characteristics from an individual hospitalization. Within an observation, three identification numbers are coded: one for the hospitalization, one for the patient, and one for the hospital in which the admission took place. The patient linkage number is used to follow patients across multiple hospitalizations within the database year, but are not unique across multiple years, which precludes following an individual patient across multiple years. Because of this, the analyses are multiple, pooled cross-sectional rather than truly longitudinal.

## Measurements and Variable Definitions

Each year contains datasets that include patient demographic data, including age (with all subjects aged > 90 aggregated as “90”), sex, payer, and information about patient’s city of residency, but do not include race or ethnicity. Variables included in the NRD and additional variables constructed for the analyses are identified in Supplemental Table 2. Income is provided in quartiles, based on ZIP code of primary residence, rather than individual actual income. Regarding insurance status, only primary payer is reported, and it should be noted that dual-eligible patients (*i.e.,* Medicaid + Medicare) will be categorized as Medicare patients (3).

Administrative data about each given hospitalization includes date of admission (serialized, rather than in calendar dates), length of stay, year, month and quarter of admission, and discharge disposition, and whether patient was a resident of the same state as the hospital where she or he received care. Resident status is included, as linkage markers are only reliably valid within the same state, potentially underestimating readmissions that occur in multiple states, possible in particular in hospitals that are near state borders. Observations where transfers to other acute care hospitals occurred are collapsed with the discharging hospital’s information reported. Discharge diagnoses and procedures are provided using ICD-9 codes for 2010 through 3^rd^ quarter of 2015, and ICD-10 for 4^th^ quarter of 2015 and all of 2016 (3).

Hospital characteristics include hospital ownership, number of beds, annual discharges, teaching status, and size of municipality where hospital is located. Notably, all those in the sample are short-term, acute care, community hospitals. Costs are provided with total filed charges and the hospitals’ cost to charge ratio for estimates. Sampling weights for each record are provided, which are calculated based on the patient and hospital characteristics relative to the overall sample in order to provide national estimates (3). The separate, annual datasets were merged, with the indicator variable for year of study retained to allow for stratification analyses by individual year. Subjects are not identifiable across individual years, though they may be present in more than one year of the study in some cases.

In Supplemental Table 2 we show an accounting of the measurements either provided by AHRQ in the database or constructed for the analysis. Full accounting of the variable specifications for those included in the NRD can be found online in the database documentation, but we have included this table for a general summary (4). Included are the description of the variable, its specifications, and the level of analysis that it represents. For continuous variables, transformations were explored using graphical laddering technique, where the distribution is plotted as a histogram and compared against a normal distribution curve, then transformed by various operations to visually determine which transformation provides the closest approximation of normality. In addition, categorical cut points into various quantiles of continuous variables were explored, with break points described in the variable explanation. For all analyses, we are using variables as specified in the index discharge, as we are looking primarily at factors that predict various readmission outcomes. References for methodology for development of constructed variables, where appropriate, are included in footnotes to the table.

## Index Hospitalization Inclusion and Exclusion Criteria

Eligible admissions include those where the patient was discharged alive and not against medical advice. In order to qualify as an index admission, there must not have been another admission for any cause in the prior thirty days (that is, a thirty-day washout period), consistent with CMS HRRP guidelines (5). Because of inability to track readmissions beyond the end of a calendar year, December hospitalizations will not be counted as index admissions. Because of the inability to determine if January hospitalizations were actually readmissions from the prior December, and to ensure that the thirty-day washout period occurred, they will also be excluded as index admissions. It should be noted that a single patient may have multiple hospitalizations over the course of the year, and as long as thirty days has elapsed since the last discharge, a new stay may be regarded as a qualifying index admission pursuant to the CMS HRRP guidelines (5).

Because of a known issue in the database, there are small fraction (<1%) of observations with overlapping stays due to miscoding of transfers (3), and as such, transfers were excluded from the analysis. As noted above, since linkages are not traceable across states, those who were not a resident of the same state as the hospital where they were admitted may have been lost to follow up when returning to their home state and were excluded. This may have resulted in under-sampling of subjects who were residents of metropolitan areas on borders or who sought care in regional referral centers. In an AHRQ analysis using the 2011 Medicare Standard Analytic File to approximate the effect of limiting to within-state admission-readmission pairs did result in a 0.41 percentage point decrease in the rates for COPD (3).

A summary of these criteria are found in Supplemental Table 3, which are adapted from the CMS HRRP program documentation and the Yale New Haven HRRP Methodology Reports (5-7).

## Readmission Specifications

Thirty-day readmissions were coded by determining the elapsed time from the index hospital discharge (determined by adding length of stay to date of admission). All cause readmissions within 30 days of the index discharge will be counted, except as for reasons excluded in the CMS HRRP policy measure documentation, with exceptions made for returns to hospital for specified diagnosis and procedure codes. (6, 7). For subjects admitted for a procedure or diagnostic reason considered a planned admission, the readmission does not qualify for this analysis and was not counted. If the subject is admitted for a procedure or diagnostic reason that is considered potentially planned, and the principal diagnosis at discharge is not an acute condition or complication of prior care, the readmission does not qualify for this analysis and was not counted. A schematic of the previously published CMS HRRP methodology for excluding planned readmissions follows below in Supplemental Figure 1.

## Additional Explanation of Statistical Methods

We fit a series of regression models in parallel in order to compare the two comorbidity indices. A naïve model was fit with just the comorbidity index of interest in order to estimate the variance components (Candidate Model 1), then we added patient-level covariates (Candidate Model 2) and hospital-level covariates (Candidate Model 3). We used two-level models (patient-stays nested in hospitals) with independent variance for the random intercepts. We did not include random slopes in the models. Information criteria were used to estimate model fit between the Elixhauser and Charlson candidate models, as when models do not include the same primary regressor and are not nested, likelihood ratio tests cannot be used to compare the two models. We used the discharge weights provided with the NRD for all analyses. The NRD provides these weights in order to calculate population estimates from the sample. The weights are derived using patient and hospital level characteristics, comparing the observed characteristics against the known possible universe of the same characteristics from national hospital survey data. A detailed accounting of how the weights are constructed is included in the NRD manual (3).

## Supplemental Tables and Figures

Supplemental Methods Table 1: Summary of included states and raw counts by year in the NRD

| Year | States Included | Number of States | Number of Hospitals | Number of Discharges (unweighted) | Number of Discharges (weighted) |
| --- | --- | --- | --- | --- | --- |
| 2010 | AK, AR, CA, FL, GA, HI, LA, MA, MO, MS, NE, NM, NY, SC, TN, UT, VA, WA | 18 | 1,809 | 13,907,610 | 37,184,093 |
| 2011 | AK, AR, CA, FL, GA, HI, LA, MA, MO, MS, NE, NM, NY, SC, TN, UT, VA, WA | 18 | 1,804 | 13,915,176 | 36,909,160 |
| 2012 | AK, AR, CA, FL, GA, HI, LA, MA, MO, NE, NM, NY, SC, TN, UT, VA, VT, WA | 18 | 1,715 | 13,459,216 | 36,465,049 |
| 2013 | AR, CA, FL, GA, HI, IA, LA, MA, MO, NE, NM, NV, NY, SC, SD, TN, UT, VA, VT, WA, WI | 21 | 2,006 | 14,325,172 | 35,580,348 |
| 2014 | AR, CA, FL, GA, HI, IA, LA, MA, MD, MO, NE, NM, NV, NY, SC, SD, TN, UT, VA, VT, WA, WI | 22 | 2,048 | 14,894,613 | 35,306,427 |
| 2015 | AK, AR, CA, FL, GA, HI, IA, LA, MA, MD, MO, MS, NE, NM, NV, NY, OR, PA, SC, SD, TN, UT, VA, VT, WA, WI, WY | 27 | 2,367 | 17,198,125 | 35,673,252 |
| 2016 | AK, AR, CA, FL, GA, HI, IA, LA, MD, MA, MI, MS, MO, NE, NV, NM, NY, OR, PA, SC, SD, TN, UT, VT, WA, WI, WY | 27 | 2,355 | 17,197,683 | 35,660,906 |
| Included in all years: | AR, CA, FL, GA, HI, LA, MA, MO, NE, NM, NY, SC, TN, UT, VA, WA | 16 | **TOTAL** | **104,897,595** | **252,779,235** |
| Adapted from NRD documentation (2) | | | | | |

Supplemental Methods Table 2: Variable construction and measurements

| Variable | Specification | Type | Level | Description |
| --- | --- | --- | --- | --- |
| **Variables provided with the NRD** | | | | |
| Observation identifier | Patient-stay ID | Observation level ID | Patient-stay | Within a given year, each individual hospitalization has a unique identifier |
| Patient identifier | Subject ID | Patient ID | Patient | Within a given year, an individual patient has a unique identifier allowing linkage between visits for readmission estimates and measures of utilization |
| Hospital identifier | Cluster ID | Clustering ID | Hospital cluster | ID variable for hospital. Since ID numbers are not traceable across years, the same hospital may be present in more than one year of the analysis but labeled as a new cluster. |
| Age | Covariate | Continuous | Patient stay | Age in years at the time of hospital discharge |
| Sex | Covariate | Categorical | Patient | Sex of patient |
| Patient residence location | Covariate | Categorical | Patient stay | Designation of patient residence at the county level by National Center for Health Statistics criteria, emphasizing urban distinctions and further dividing between central and fringe counties in major metropolitan areas, with smaller metropolitan areas divided by population size, and non-metropolitan areas divided into micropolitan and non-core (rural) categories |
| Patient income | Covariate | Categorical | Patient stay | Classification of the estimated median household income of residents in the patient's ZIP code, provided in quartile form by AHRQ in the database. This data is derived from demographic data from the Claritas database. Notably, values are coded as missing in this field if not available, or in instances with ZIP codes having populations below a minimum threshold, or if only one ZIP code within a quartile was present in a given state to protect confidentiality^1^ |
| Year | Fixed effect | Categorical | Patient stay | Calendar year of discharge from index hospitalization |
| Discharge Quarter | Fixed effect | Categorical | Patient stay | Quarter of discharge from index hospitalization |
| Discharge Month | Fixed effect | Categorical | Patient stay | Month of discharge from index hospitalization |
| Length of stay | Covariate | Continuous | Patient stay | Length of stay in whole days of index hospitalization |
| Primary payer | Covariate | Categorical | Patient stay | Expected primary payer at time of discharge. Includes Medicare (includes managed and fee-for-service), Medicaid (includes managed and fee-for-service), private (includes HMO and PPO, commercial carriers), self-pay, no charge, and other categories (includes worker's compensation and other government programs) |
| Discharge disposition | Covariate | Categorical | Patient stay | Disposition at time of discharge from index hospitalization. Includes routine discharges home without services, transfers to other short-term hospitals for further inpatient care, transfers to other health facilities (including skilled nursing, intermediate care, hospice, psychiatric hospitals, and long-term acute care hospitals), discharges home with home health services (including home hospice), transfer to law enforcement, or discharged alive but destination unknown. For this analysis, patients who left against medical advice or died during index hospitalization are excluded, in line with CMS HRRP policy. Will be broken down further into dummy variables for (a) home without services, (b) home with home health, (c) transfer for post-acute care services |
| Hospital ownership | Covariate | Categorical | Hospital cluster | Ownership or control of hospital, includes government/nonfederal (county or municipal, non-Veterans Affairs facilities), private/non-profit, or private/for-profit designations |
| Hospital bed size | Covariate | Categorical | Hospital cluster | Size of hospital by beds grouped into small/medium/large., The cutoffs for each category of which vary by region.^1^ |
| Hospital teaching status | Covariate | Categorical | Hospital cluster | Teaching status of hospital, divided into metropolitan/non-teaching, metropolitan/non-teaching, or non-metropolitan (which are considered non-teaching in this database due to the very low frequency of rural teaching hospitals). For models, this was collapsed into teaching versus non-teaching. |
| Hospital urban/rural designation | Covariate | Categorical | Hospital cluster | Designation of hospital location at the county level as provided by American Hospital Association files. Includes large metropolitan (≥1 million residents), small metropolitan (<1 million residents), micropolitan, and non-urban categories. |
| **Constructed Variables** | | | | |
| Charlson Comorbidity Index Score | Primary Predictor | Continuous | Patient-stay | Summary score of Charlson Comorbidity Index at time of hospital discharge, tested as continuous and categorial (by quartile) score levels, tabulated from ICD codes^2^ and score weighted per Charlson methodology^3^ |
| Elixhauser Comorbidity Index Score | Primary Predictor | Continuous | Patient stay | Summary score of Elixhauser Comorbidity Index at time of hospital discharge, tested as continuous and categorial (by quartile) score levels, tabulated from ICD codes^2^ and score weighted per Elixhauser methodology^4^ |
| Individual comorbid conditions | Covariate | Indicator | Patient stay | Individual comorbidity flags from Elixhauser and Charlson scores retained for sub-analyses |
| Use of non-invasive ventilation | Covariate | Indicator | Patient stay | Use of non-invasive ventilation (CPAP or BPAP) during index hospitalization by ICD codes |
| Use of mechanical ventilation | Covariate | Indicator | Patient stay | Intubation and/or use of mechanical ventilation during index hospitalization  by ICD codes |
| Placement or presence of tracheostomy | Covariate | Indicator | Patient stay | Placement of tracheostomy or presence of long-term tracheostomy during index hospitalization  by ICD codes |
| Occurrence of cardiac arrest | Covariate | Indicator | Patient stay | Occurrence of cardiac arrest during index hospitalization by ICD codes |
| Performance of resuscitation | Covariate | Indicator | Patient stay | Performance of cardiopulmonary resuscitation during index hospitalization by ICD codes |
| Use of extra-corporeal life support | Covariate | Indicator | Patient stay | Use of extra-corporeal membrane oxygenation (ECMO) during index hospital stay by ICD codes |
| Hospital COPD discharge volume | Covariate | Continuous | Hospital cluster | Number of qualifying COPD index discharges in the analysis year |
| Proportion of Medicaid patient-days estimate | Covariate | Continuous | Hospital cluster | Proxy for safety net hospital status, defined as proportion of patient days in hospital with expected payer of Medicaid |
| Utilization estimate | Covariate | Continuous | Patient | Estimate of utilization status, defined as number of overall hospital stays for any cause over the course of a given year for a single patient |
| Scatter of care | Covariate | Continuous | Patient | Estimate of care fragmentation, defined as number of hospitals in which patient sought care in the given year |
| ^1^ AHRQ NRD Database Documentation (3)  ^2^ Adapted Charlson and Elixhauser Stata Macros (8, 9)  ^3^ Charlson scores weighted per original derivation (10)  ^4^ Elixhauser readmission weighted scores per AHRQ version (11) | | | | |

Supplemental Table 3: Criteria for inclusion and exclusion as an index COPD hospitalization

| **Inclusion Criteria** | **Rationale** |
| --- | --- |
| Discharge from hospital for acute exacerbation of COPD as principal discharge diagnosis | Coding algorithm designed as specified in Published HRRP methodology^1^ |
| Discharge from hospital for acute respiratory failure as principal discharge diagnosis with COPD as secondary diagnosis | Coding algorithm designed as specified in Published HRRP methodology^1^ |
|  |  |
| **Exclusion Criteria** | **Rationale** |
| Discharges in January | Unable to determine if stay is a readmission from previous December due to multiple cross-sectional rather than longitudinal database^2^ |
| Discharges in December | Unable to follow for readmission into following January across calendar years due to multiple cross-sectional rather than longitudinal database^2^ |
| Discharge within 30 days of a previous admission for any cause | Published HRRP methodology^1^ includes a wash out period of 30 days before another hospitalization qualifies as an index stay |
| Discharges against medical advice | Published HRRP methodology^1^ excludes these cases as care coordination limited by patient leaving |
| Death in index hospital stay | Unable to be readmitted if died during first admission^1^ |
| Discharge from hospital with fewer than 25 COPD index admissions in a given year | Published HRRP methodology^1^ excludes these cases for lack of volume of condition of interest |
| Discharge from hospital in state other than that of patient residency | Construction of database limits ability to follow patient across state lines due to non-unique identifiers^2^ |
| Transfer to other facility same day as admission | Inconsistencies of coding transfers^2^ complicated the analysis, with small number, judgment call made to drop these stays to simplify coding |
| ^1^ HRRP methodology reports (6, 7)  ^2^ AHRQ NRD Database Documentation (3) | |


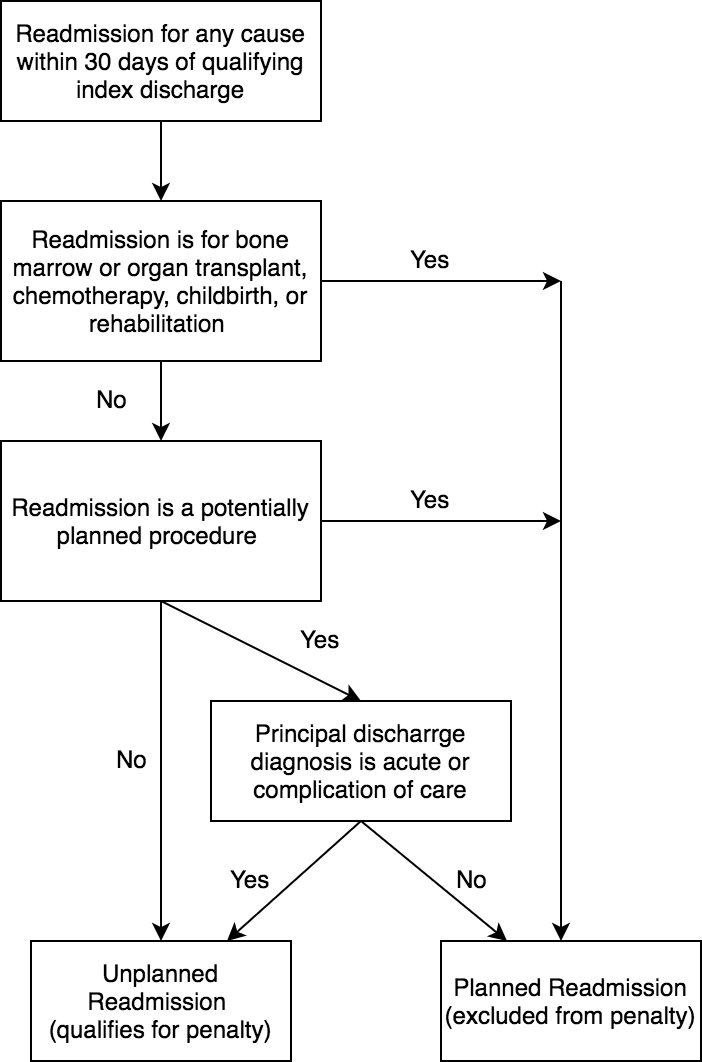


Supplemental Figure 1: Published HRRP schema for excluding planned readmissions from the analysis. Figure adapted from Yale New Haven HRRP Methodology Reports, where diagnosis and procedure codes for exceptions can be found (6, 7).

## Supplement References

1. HCUP Nationwide Readmissions Database (NRD) [Internet]. Agency for Healthcare Research and Quality. 2010-2016. Available from: https://www.hcup-us.ahrq.gov/nrdoverview.jsp.

2. Healthcare Cost and Utilization Project. NRD Summary Statistics Rockville, MD: Agency for Healthcare Research and Quality; 2017 [updated November 2017. Available from: https://www.hcup-us.ahrq.gov/db/nation/nrd/nrdsummstats.jsp.

3. Healthcare Cost and Utilization Project. Introduction to the HCUP Nationwide Readmissions Database (NRD) 2010-2016 Rockville, MD: Agency for Healthcare Research and Quality; 2018 [updated August 2018. Available from: https://www.hcup-us.ahrq.gov/db/nation/nrd/Introduction_NRD_2010-2016.jsp.

4. Healthcare Cost and Utilization Project. NRD Description of Data Elements Rockville, MD: Agency for Healthcare Research and Quality; 2018 [updated August 2018. Available from: https://www.hcup-us.ahrq.gov/db/nation/nrd/nrddde.jsp.

5. Centers for Medicare & Medicaid Services. Readmissions Reduction Program 2017 [updated 30 November 2017. Available from: https://www.cms.gov/Medicare/Medicare-Fee-for-Service-Payment/AcuteInpatientPPS/Readmissions-Reduction-Program.html.

6. Yale New Haven Health Services Corporation/Center for Outcomes Research & Evaluation. 2016 Condition-Specific Measures Updates and Specifications Report Hospital-Level 30-Day Risk-Standardized Readmission Measures. Baltimore, MD: Centers for Medicare & Medicaid Services; 2016.

7. Yale New Haven Health Services Corporation/Center for Outcomes Research & Evaluation. 2017 Condition-Specific Measures Updates and Specifications Report Hospital-Level 30-Day Risk-Standardized Readmission Measures. Baltimore, MD: Centers for Medicare & Medicaid Services; 2017.

8. Stagg V. CHARLSON: Stata module to calculate Charlson index of comorbidity. Boston College Department of Economics: Statistical Software Components; 2006.

9. Stagg V. ELIXHAUSER: Stata module to calculate Elixhauser index of comorbidity. Boston College Department of Economics: Statistical Software Components; 2015.

10. Charlson M, Szatrowski TP, Peterson J, Gold J. Validation of a combined comorbidity index. J Clin Epidemiol. 1994;47(11):1245-51.

11. Moore BJ, White S, Washington R, Coenen N, Elixhauser A. Identifying Increased Risk of Readmission and In-hospital Mortality Using Hospital Administrative Data: The AHRQ Elixhauser Comorbidity Index. Med Care. 2017;55(7):698-705.
