# Supplementary figures and images for: Comorbidity and thirty-day hospital readmission odds in chronic obstructive pulmonary disease: a comparison of the Charlson and Elixhauser comorbidity indices
Source: BMC Health Serv Res. 2019 Oct 15;19:701. doi: 10.1186/s12913-019-4549-4 (PMC6794890; doi:10.1186/s12913-019-4549-4)

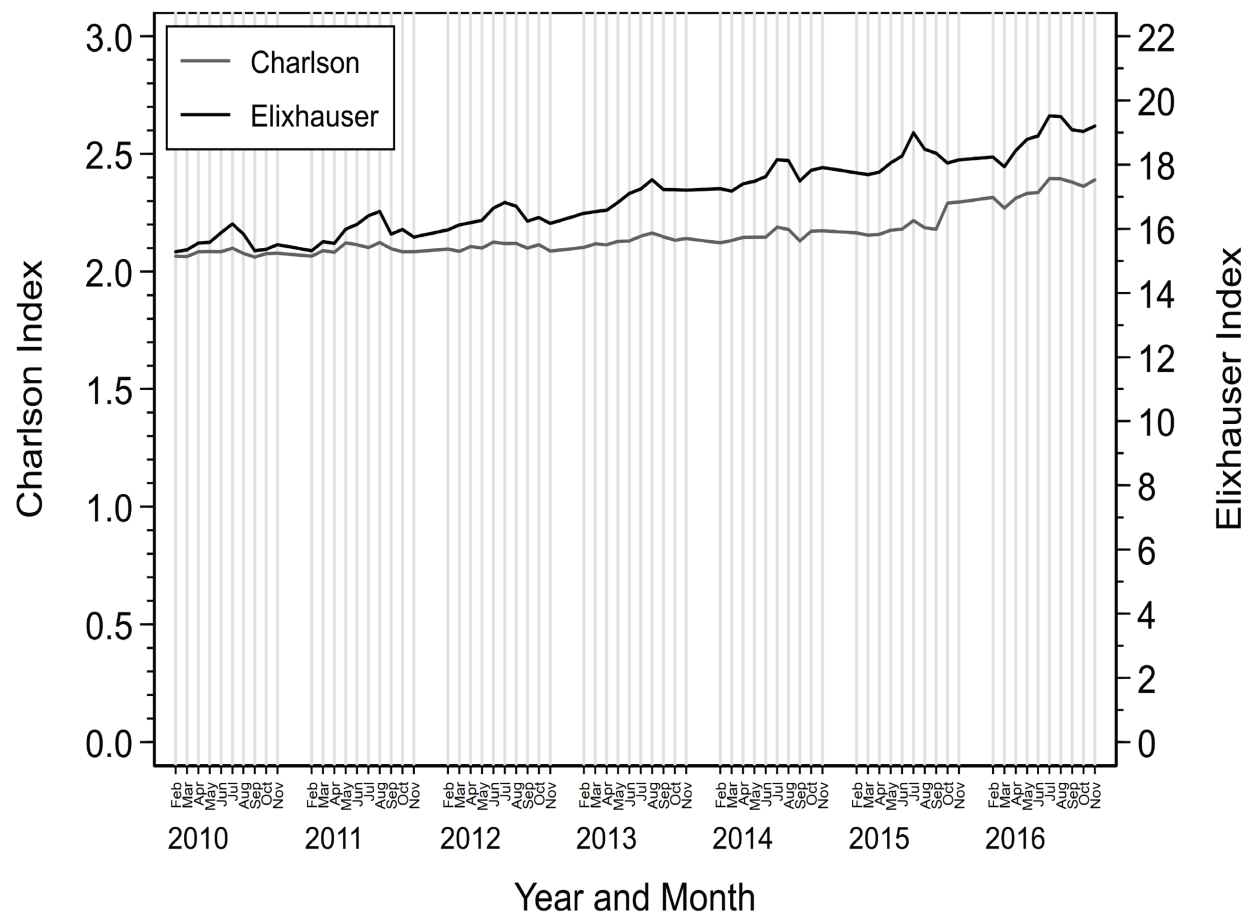

Supplemental Figure: Trend of mean Charlson and Elixhauser Index scores over time

Supplement: Supplementary file 2 — Additional file 2: Figure S1. Trend of mean Charlson and Elixhauser Index scores over time. [file 12913_2019_4549_MOESM2_ESM.pdf]
