# Supplementary material for: Comorbidity and thirty-day hospital readmission odds in chronic obstructive pulmonary disease: a comparison of the Charlson and Elixhauser comorbidity indices
Source: BMC Health Serv Res. 2019 Oct 15;19:701. doi: 10.1186/s12913-019-4549-4 (PMC6794890; doi:10.1186/s12913-019-4549-4)
Supplement: Supplementary file 3 — Additional file 3: Table S1. Multilevel Logistic Regression models of Readmission using Charlson Index using Hospital Level random intercept. [file 12913_2019_4549_MOESM3_ESM.pdf]

Supplemental Table: Multilevel Logistic Regression models of Readmission using Charlson Index using Hospital Level random intercept

Model Info	Model 1		Model 2		Model 3	
N	1,662,983		1,659,576		1,658,372	
LL	-1,702,361.6		-1,693,222.5		-1,683,418.1	
df	3		22		41	
AIC	3,404,729.3		3,386,489.0		3,366,918.3	
BIC	3,404,766.3		3,386,760.0		3,367,423.5	
**Predictors**	**OR (95% CI)**	***P***	**OR (95% CI)**	***P***	**OR (95% CI)**	***P***
**Charlson Index** *(per 1)*	1.15 (1.15, 1.15)	<.001	1.14 (1.13, 1.14)	<.001	1.12 (1.12, 1.12)	<.001
**Year** *(ref=2010)*						
2011			1.00 (0.97, 1.02)	0.709	1.00 (0.97, 1.02)	0.673
2012			0.96 (0.94, 0.98)	<.001	0.96 (0.94, 0.98)	<.001
2013			0.93 (0.91, 0.95)	<.001	0.93 (0.91, 0.95)	<.001
2014			0.92 (0.90, 0.94)	<.001	0.92 (0.90, 0.94)	<.001
2015			0.88 (0.87, 0.90)	<.001	0.88 (0.86, 0.90)	<.001
2016			0.88 (0.86, 0.90)	<.001	0.87 (0.85, 0.89)	<.001
**Quarter** *(ref=1st)*						
2nd Quarter			0.97 (0.95, 0.98)	<.001	0.97 (0.96, 0.98)	<.001
3rd Quarter			1.01 (0.99, 1.02)	0.411	1.01 (1.00, 1.02)	0.167
4th Quarter			0.99 (0.97, 1.00)	0.038	0.99 (0.98, 1.01)	0.277
**Sex** *(ref=male)*						
Female			0.94 (0.93, 0.95)	<.001	0.92 (0.91, 0.93)	<.001
**Age** *(per 10 year)*			1.00 (0.99, 1.00)	0.796	0.97 (0.97, 0.98)	<.001
**Income Quartile** *(ref=1st)*						
2nd Quartile			0.98 (0.97, 1.00)	0.019	0.98 (0.97, 0.99)	0.003
3rd Quartile			0.98 (0.97, 1.00)	0.015	0.97 (0.95, 0.98)	<.001
4th Quartile			0.97 (0.95, 0.99)	<.001	0.94 (0.93, 0.96)	<.001
Missing			0.96 (0.93, 1.00)	0.073	0.97 (0.93, 1.00)	0.083
**Payer** *(ref=Medicare)*						
Medicaid			1.07 (1.05, 1.09)	<.001	1.08 (1.06, 1.10)	<.001
Private			0.67 (0.66, 0.68)	<.001	0.69 (0.68, 0.71)	<.001
Self-Pay			0.59 (0.57, 0.61)	<.001	0.62 (0.60, 0.64)	<.001
Other/No Charge			0.77 (0.75, 0.80)	<.001	0.79 (0.77, 0.82)	<.001
**Disposition** *(ref=Routine to home)*						
Post-acute care					1.30 (1.28, 1.32)	<.001
Other					1.13 (1.06, 1.20)	<.001
Home Health					1.35 (1.33, 1.37)	<.001
**Length of Stay** *(per day)*					1.02 (1.02, 1.02)	<.001
**Care intensity** *(ref=No)*						
Non-invasive ventilation					1.16 (1.14, 1.18)	<.001
Mechanical ventilation					0.95 (0.92, 0.97)	<.001
Tracheostomy					1.02 (0.97, 1.09)	0.425
Cardiac arrest					0.82 (0.74, 0.91)	<.001
CPR					1.08 (0.95, 1.24)	0.242
**Hospital ownership** *(ref=government)*						
Private, non-profit					0.99 (0.98, 1.01)	0.437
Private, for-profit					1.04 (1.01, 1.06)	<.001
**Hospital teaching status** *(ref=Non-teaching)*						
Teaching Hospital					1.01 (0.99, 1.02)	0.423
**Hospital location** *(ref=Large metro area)*						
Small metro area					0.94 (0.92, 0.95)	<.001

Micropolitan area	0.89 (0.87, 0.91)	<.001
Rural	0.87 (0.84, 0.90)	<.001
**Hospital Bed Size** *(ref=Small)*		
Medium	1.01 (0.99, 1.03)	0.174
Large	1.02 (1.00, 1.04)	0.040
**Annual Discharge (per 10k)**	1.01 (1.00, 1.02)	0.130
**Proportion Medicaid per 10%**	1.00 (1.00, 1.01)	0.531
