# Supplementary material for: Comorbidity and thirty-day hospital readmission odds in chronic obstructive pulmonary disease: a comparison of the Charlson and Elixhauser comorbidity indices
Source: BMC Health Serv Res. 2019 Oct 15;19:701. doi: 10.1186/s12913-019-4549-4 (PMC6794890; doi:10.1186/s12913-019-4549-4)
Supplement: Supplementary file 5 — Additional file 5: Table S3. Charlson and Elixhauser Indices (with 95% CI) over time per 1/2 standard deviation increase. [file 12913_2019_4549_MOESM5_ESM.pdf]

Supplemental Table: Charlson and Elixhauser Indices (with 95% CI) over time per ½ standard deviation increase

| | 2010 | 2011 | 2012 | 2013 | 2014 | 2015 | 2016 |
|---|---|---|---|---|---|---|---|
| **Charlson** | | | | | | | |
| Model 1 | 1.11 (1.11, 1.12) | 1.11 (1.10, 1.12) | 1.11 (1.10, 1.12) | 1.11 (1.10, 1.12) | 1.11 (1.10, 1.12) | 1.11 (1.11, 1.12) | 1.11 (1.10, 1.11) |
| Model 2 | 1.10 (1.09, 1.11) | 1.10 (1.09, 1.11) | 1.10 (1.09, 1.11) | 1.10 (1.09, 1.11) | 1.10 (1.09, 1.11) | 1.11 (1.10, 1.11) | 1.10 (1.09, 1.10) |
| Model 3 | 1.09 (1.08, 1.10) | 1.09 (1.08, 1.10) | 1.09 (1.08, 1.09) | 1.09 (1.08, 1.10) | 1.09 (1.08, 1.10) | 1.10 (1.09, 1.10) | 1.09 (1.08, 1.09) |
| **Elixhauser** | | | | | | | |
| Model 1 | 1.16 (1.15, 1.17) | 1.16 (1.15, 1.17) | 1.15 (1.14, 1.15) | 1.15 (1.14, 1.16) | 1.15 (1.14, 1.16) | 1.15 (1.14, 1.16) | 1.15 (1.14, 1.16) |
| Model 2 | 1.14 (1.14, 1.15) | 1.15 (1.14, 1.16) | 1.14 (1.13, 1.14) | 1.14 (1.13, 1.15) | 1.14 (1.13, 1.15) | 1.14 (1.14, 1.15) | 1.14 (1.14, 1.15) |
| Model 3 | 1.13 (1.12, 1.14) | 1.13 (1.12, 1.14) | 1.12 (1.11, 1.13) | 1.12 (1.12, 1.13) | 1.12 (1.11, 1.13) | 1.13 (1.12, 1.14) | 1.12 (1.12, 1.13) |

Model 2 adjusted for age, sex, income, time period (year and quarter), and insurer.
Model 3 adjusted for Model 2 covariates as well as discharge disposition, hospital length of stay, care intensity, and hospital characteristics (ownership type, teaching hospital status, location, size, annual number of discharges, and proportion Medicaid patients).
