# Supplementary material for: Comorbidity and thirty-day hospital readmission odds in chronic obstructive pulmonary disease: a comparison of the Charlson and Elixhauser comorbidity indices
Source: BMC Health Serv Res. 2019 Oct 15;19:701. doi: 10.1186/s12913-019-4549-4 (PMC6794890; doi:10.1186/s12913-019-4549-4)
Supplement: Supplementary file 6 — Additional file 6: Figure S2. Changes in Charlson and Elixhauser Indices (with 95% CI) overtime per 1/2 standard deviation increase. [file 12913_2019_4549_MOESM6_ESM.pdf]

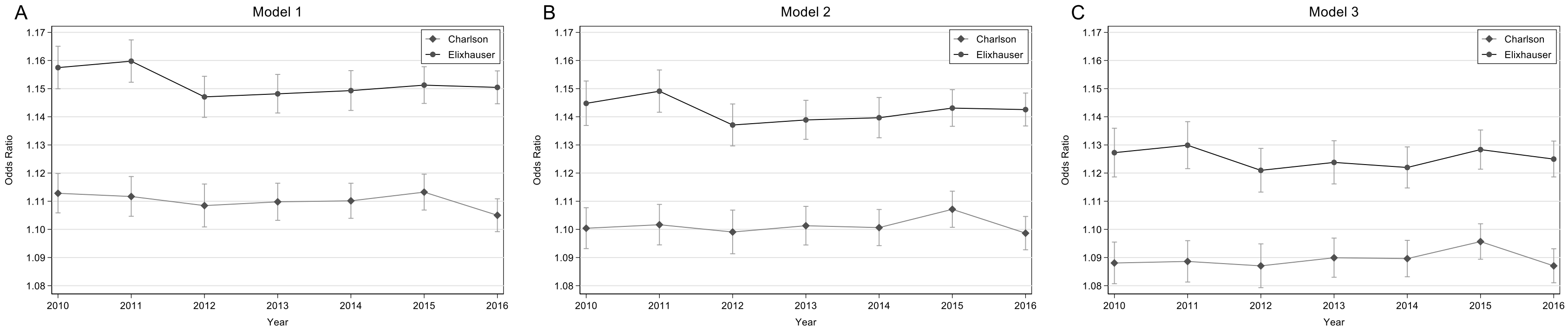

Supplemental Figure 4: Changes in Charlson and Elixhauser Indices (with 95% CI) overtime per ½ standard deviation increase. Model 1 contains only comorbidity index. Model 2 adjusted for age, sex, income, time period (year and quarter), and insurer. Model 3 adjusted for Model 2 covariates as well as discharge disposition, hospital length of stay, care intensity, and hospital characteristics (ownership type, teaching hospital status, location, size, annual number of discharges, and proportion of Medicaid patients).
