# Supplementary material for: Comorbidity and thirty-day hospital readmission odds in chronic obstructive pulmonary disease: a comparison of the Charlson and Elixhauser comorbidity indices
Source: BMC Health Serv Res. 2019 Oct 15;19:701. doi: 10.1186/s12913-019-4549-4 (PMC6794890; doi:10.1186/s12913-019-4549-4)
Supplement: Supplementary file 8 — Additional file 8: Table S5. Multilevel Logistic Regression models of Readmission using Elixhauser Index using Hospital Level random intercept (age 18 and older). [file 12913_2019_4549_MOESM8_ESM.pdf]

Supplemental Table: Multilevel Logistic Regression models of Readmission using Elixhauser Index using Hospital Level random intercept (age 18 and older)

**Model Info**	**Model 1**		**Model 2**		**Model 3**	
N	1,682,629		1,679,169		1,677,941	
LL	-1,709,883.6		-1,701,310.5		-1,695,230.0	
df	3		22		41	
AIC	3,419,773.3		3,402,665.0		3,390,542.0	
BIC	3,419,810.3		3,402,936.3		3,391,047.8	
**Predictors**	**OR (95% CI)**	***P***	**OR (95% CI)**	***P***	**OR (95% CI)**	***P***
**Elixhauser Index** *(per 3)*	1.06 (1.06, 1.06)	<.001	1.05 (1.05, 1.06)	<.001	1.05 (1.05, 1.05)	<.001
**Year** *(ref=2010)*						
2011			0.99 (0.97, 1.01)	0.469	0.99 (0.97, 1.01)	0.432
2012			0.95 (0.93, 0.97)	<.001	0.95 (0.93, 0.97)	<.001
2013			0.91 (0.89, 0.93)	<.001	0.91 (0.89, 0.93)	<.001
2014			0.89 (0.87, 0.91)	<.001	0.89 (0.87, 0.90)	<.001
2015			0.86 (0.84, 0.88)	<.001	0.86 (0.84, 0.88)	<.001
2016			0.86 (0.84, 0.88)	<.001	0.85 (0.83, 0.87)	<.001
**Quarter** *(ref=1st)*						
2nd Quarter			0.96 (0.95, 0.98)	<.001	0.97 (0.95, 0.98)	<.001
3rd Quarter			1.00 (0.98, 1.01)	0.618	1.00 (0.99, 1.01)	0.912
4th Quarter			0.98 (0.97, 1.00)	0.008	0.99 (0.97, 1.00)	0.044
**Sex** *(ref=male)*						
Female			0.93 (0.93, 0.94)	<.001	0.92 (0.91, 0.92)	<.001
**Age** *(per 10 year)*			1.01 (1.00, 1.01)	<.001	0.99 (0.98, 0.99)	<.001
**Income Quartile** *(ref=1st)*						
2nd Quartile			0.98 (0.97, 1.00)	0.033	0.98 (0.97, 1.00)	0.008
3rd Quartile			0.98 (0.97, 1.00)	0.012	0.97 (0.95, 0.98)	<.001
4th Quartile			0.97 (0.95, 0.99)	<.001	0.95 (0.93, 0.97)	<.001
Missing			0.97 (0.93, 1.01)	0.164	0.97 (0.93, 1.01)	0.156
**Payer** *(ref=Medicare)*						
Medicaid			1.06 (1.05, 1.08)	<.001	1.07 (1.05, 1.09)	<.001
Private			0.70 (0.68, 0.71)	<.001	0.71 (0.70, 0.72)	<.001
Self-Pay			0.61 (0.59, 0.63)	<.001	0.63 (0.61, 0.65)	<.001
Other/No Charge			0.78 (0.76, 0.81)	<.001	0.80 (0.77, 0.82)	<.001
**Disposition** *(ref=Routine to home)*						
Post-acute care					1.20 (1.18, 1.22)	<.001
Other					1.07 (1.00, 1.14)	0.037
Home Health					1.30 (1.28, 1.32)	<.001
**Length of Stay** *(per day)*					1.01 (1.01, 1.01)	<.001
**Care intensity** *(ref=No)*						
Non-invasive ventilation					1.09 (1.07, 1.11)	<.001
Mechanical ventilation					0.82 (0.79, 0.84)	<.001
Tracheostomy					1.05 (0.99, 1.11)	0.114
Cardiac arrest					0.82 (0.74, 0.90)	<.001
CPR					1.08 (0.95, 1.24)	0.233
**Hospital ownership** *(ref=government)*						
Private, non-profit					0.98 (0.96, 1.00)	0.017
Private, for-profit					1.03 (1.01, 1.06)	0.001
**Hospital teaching status** *(ref=Non-teaching)*						
Teaching Hospital					1.00 (0.99, 1.02)	0.937
**Hospital location** *(ref=Large metro area)*						
Small metro area					0.93 (0.92, 0.95)	<.001

Micropolitan area	0.90 (0.88, 0.92)	<.001
Rural	0.89 (0.86, 0.92)	<.001
**Hospital Bed Size** *(ref=Small)*		
Medium	1.01 (0.99, 1.03)	0.447
Large	1.01 (0.99, 1.03)	0.463
**Annual Discharge (per 10k)**	1.01 (1.00, 1.03)	0.008
**Proportion of Medicaid per 10%**	1.00 (0.99, 1.01)	0.840
