# Supplementary material for: Comorbidity and thirty-day hospital readmission odds in chronic obstructive pulmonary disease: a comparison of the Charlson and Elixhauser comorbidity indices
Source: BMC Health Serv Res. 2019 Oct 15;19:701. doi: 10.1186/s12913-019-4549-4 (PMC6794890; doi:10.1186/s12913-019-4549-4)
Supplement: Supplementary file 9 — Additional file 9: Table S6. Multilevel Logistic Regression models of Readmission using only the covariates as predictor with Hospital Level random intercept. [file 12913_2019_4549_MOESM9_ESM.pdf]

Supplemental Table: Multilevel Logistic Regression models of Readmission using only the covariates as predictor with Hospital Level random intercept

Model Info	Model 1	Model 2		Model 3	
N		1,659,576		1,658,372	
LL		-761,221.6		-755,666.8	
df		20		39	
AIC		1,522,483.1		1,511,411.5	
BIC		1,522,729.5		1,511,892.1	
**Predictors**		**OR (95% CI)**	***P***	**OR (95% CI)**	***P***
**Year** *(ref=2010)*					
2011		1.00 (0.99, 1.02)	0.893	1.00 (0.99, 1.02)	0.933
2012		0.98 (0.96, 0.99)	0.002	0.97 (0.96, 0.99)	<.001
2013		0.95 (0.94, 0.97)	<.001	0.94 (0.93, 0.96)	<.001
2014		0.94 (0.92, 0.95)	<.001	0.95 (0.93, 0.96)	<.001
2015		0.93 (0.91, 0.94)	<.001	0.92 (0.90, 0.93)	<.001
2016		0.93 (0.92, 0.95)	<.001	0.92 (0.90, 0.93)	<.001
**Quarter** *(ref=1st)*					
2nd Quarter		0.98 (0.97, 0.99)	<.001	0.98 (0.97, 0.99)	<.001
3rd Quarter		1.03 (1.02, 1.04)	<.001	1.03 (1.02, 1.04)	<.001
4th Quarter		1.00 (0.99, 1.01)	0.847	1.01 (0.99, 1.02)	0.296
**Sex** *(ref=male)*					
Female		0.91 (0.91, 0.92)	<.001	0.89 (0.89, 0.90)	<.001
**Age** *(per 10 year)*		1.02 (1.02, 1.03)	<.001	0.99 (0.98, 0.99)	<.001
**Income Quartile** *(ref=1st)*					
2nd Quartile		0.98 (0.97, 0.99)	<.001	0.97 (0.96, 0.98)	<.001
3rd Quartile		0.98 (0.96, 0.99)	<.001	0.95 (0.94, 0.96)	<.001
4th Quartile		0.99 (0.98, 1.01)	0.28	0.94 (0.93, 0.95)	<.001
Missing		0.93 (0.90, 0.97)	<.001	0.94 (0.91, 0.97)	<.001
**Payer** *(ref=Medicare)*					
Medicaid		1.07 (1.06, 1.09)	<.001	1.07 (1.06, 1.09)	<.001
Private		0.65 (0.64, 0.66)	<.001	0.68 (0.67, 0.69)	<.001
Self-Pay		0.57 (0.55, 0.58)	<.001	0.60 (0.59, 0.62)	<.001
Other/No Charge		0.75 (0.73, 0.77)	<.001	0.78 (0.76, 0.80)	<.001
**Disposition** *(ref=Routine to home)*					
Post-acute care				1.32 (1.31, 1.34)	<.001
Other				1.18 (1.13, 1.24)	<.001
Home Health				1.37 (1.36, 1.38)	<.001
**Length of Stay** *(per day)*				1.02 (1.02, 1.02)	<.001
**Care intensity** *(ref=No)*					
Non-invasive ventilation				1.17 (1.15, 1.19)	<.001
Mechanical ventilation				0.97 (0.95, 0.99)	<.001
Tracheostomy				1.00 (0.95, 1.04)	0.867
Cardiac arrest				0.84 (0.77, 0.92)	<.001
CPR				1.03 (0.93, 1.15)	0.581
**Hospital ownership** *(ref=government)*					
Private, non-profit				1.00 (0.99, 1.01)	0.784
Private, for-profit				1.03 (1.01, 1.04)	0.001
**Hospital teaching status** *(ref=Non-teaching)*					
Teaching Hospital				1.01 (1.00, 1.02)	0.192
**Hospital location** *(ref=Large metro area)*					
Small metro area				0.94 (0.93, 0.94)	<.001
Micropolitan area				0.90 (0.88, 0.91)	<.001

Rural	0.87 (0.85, 0.89)	<.001
**Hospital Bed Size** *(ref=Small)*		
Medium	1.01 (1.00, 1.03)	0.038
Large	1.02 (1.01, 1.04)	0.002
**Annual Discharge (per 10k)**	1.01 (1.00, 1.02)	<.001
**Proportion of Medicaid per 10%**	1.00 (1.00, 1.00)	0.861
