# Supplementary material for: Comorbidity and thirty-day hospital readmission odds in chronic obstructive pulmonary disease: a comparison of the Charlson and Elixhauser comorbidity indices
Source: BMC Health Serv Res. 2019 Oct 15;19:701. doi: 10.1186/s12913-019-4549-4 (PMC6794890; doi:10.1186/s12913-019-4549-4)
Supplement: Supplementary file 10 — Additional file 10: Table S7. Comparisons between covariate-only and comorbidity index models. [file 12913_2019_4549_MOESM10_ESM.pdf]

Supplemental Table: Comparisons between covariate-only and comorbidity index models

		Model 2		Model 3	
**Index Type**		**LL**	**DF**	**LL**	**DF**
	Covariate Only	-1,702,657.30	21	-1,690,687.40	40
Charlson Index	With Index	-1,693,222.5	22	-1,683,418.1	41
	-2LL Δ	18,869.6	1	14,538.6	1
	Covariate Only	-1,702,657.30	21	-1,690,687.40	40
Elixhauser Index	With Index	-1,683,905.4	22	-1,677,856.3	41
	-2LL Δ	37,503.8	1	25,662.2	1
