# Supplementary material for: Comorbidity and thirty-day hospital readmission odds in chronic obstructive pulmonary disease: a comparison of the Charlson and Elixhauser comorbidity indices
Source: BMC Health Serv Res. 2019 Oct 15;19:701. doi: 10.1186/s12913-019-4549-4 (PMC6794890; doi:10.1186/s12913-019-4549-4)
Supplement: Supplementary file 11 — Additional file 11: Table S8. Pooled cohort characteristics of patients, stratified by hospital teaching status. [file 12913_2019_4549_MOESM11_ESM.pdf]

*Supplemental Table: Pooled cohort characteristics of patients, stratified by hospital teaching status.*

| | Not Readmitted | | Readmitted | | *P* |
|---|---|---|---|---|---|
| | Non-Teaching N=816,734 | Teaching N=558,365 | Non-Teaching N=167,891 | Teaching N=119,993 | |
| **Sex**, *%* | | | | | |
| Male | 40.7% | 40.9% | 42.8% | 42.7% | <.001 |
| Female | 59.3% | 59.1% | 57.2% | 57.3% | |
| **Age**, *Mean ± SD* | 68.1 ± 11.9 | 67.6 ± 12.0 | 69.0 ± 11.7 | 68.3 ± 11.8 | <.001 |
| **Median household income by ZIP code**, *%* | | | | | |
| 1st Quartile | 38.2% | 35.2% | 38.3% | 36.3% | <.001 |
| 2nd Quartile | 28.5% | 24.2% | 28.1% | 24.0% | |
| 3rd Quartile | 19.8% | 22.7% | 19.8% | 22.1% | |
| 4th Quartile | 11.8% | 16.8% | 12.3% | 16.5% | |
| Missing | 1.6% | 1.1% | 1.6% | 1.1% | |
| [1]**Patient geographic location**, *%* | | | | | |
| Central county metro area ≥1M | 13.6% | 34.4% | 14.5% | 36.2% | <.001 |
| Fringe county metro area ≥1M | 24.3% | 24.6% | 26.0% | 24.9% | |
| County metro area 250,000-999,999k | 17.3% | 26.2% | 17.1% | 25.0% | |
| County metro area 50,000-249,999k | 11.2% | 9.1% | 11.2% | 8.6% | |
| Micropolitan area | 20.5% | 2.3% | 19.2% | 2.2% | |
| Non-metro/non-micropolitan (rural) | 13.1% | 3.4% | 12.0% | 3.1% | |
| [2]**Primary Payer**, *%* | | | | | |
| Medicare (includes dual-eligible) | 70.7% | 67.9% | 75.6% | 72.5% | <.001 |
| Medicaid | 10.5% | 13.6% | 11.6% | 15.0% | |
| Private insurance | 12.4% | 12.1% | 8.5% | 8.1% | |
| Self-pay | 3.4% | 3.4% | 1.9% | 2.0% | |
| Other, including no-charge | 3.1% | 3.0% | 2.5% | 2.3% | |
| **Number of admissions each patient had over a year**, *Mean ± SD* | 2.10 ± 1.56 | 2.16 ± 1.64 | 4.23 ± 2.42 | 4.42 ± 2.60 | <.001 |
| **Number hospitals where each patient received care over a year**, *Mean ± SD* | 1.31 ± 0.64 | 1.31 ± 0.65 | 1.43 ± 0.74 | 1.45 ± 0.76 | <.001 |
| **Discharge disposition**, *%* | | | | | |
| Routine to home | 69.9% | 67.7% | 60.7% | 59.2% | <.001 |
| Transfer to post-acute care | 12.4% | 12.4% | 16.3% | 16.2% | |
| Home with home health services | 0.9% | 0.6% | 0.9% | 0.6% | |
| Other | 16.8% | 19.3% | 22.0% | 24.1% | |
| [§]**Length of Stay**, *Mean ± SD* | 3.64 ± 1.88 | 3.71 ± 2.09 | 4.12 ± 2.28 | 4.20 ± 2.51 | <.001 |
| **Care intensity and complications**, *%* | | | | | |
| Use of non-invasive ventilation | 6.8% | 8.9% | 8.8% | 10.9% | <.001 |
| Use of mechanical ventilation | 4.1% | 5.1% | 5.3% | 6.2% | <.001 |
| Placement or presents of tracheostomy | 0.6% | 1.0% | 0.9% | 1.5% | <.001 |
| Cardiac arrest | 0.2% | 0.2% | 0.2% | 0.3% | <.001 |
| Performance of CPR | 0.1% | 0.1% | 0.2% | 0.2% | <.001 |

Note: Unweighted N's displayed. Frequencies derived using weighted analysis. Continuous variables compared by Welch's T-test and categorical variables by ANOVA.

[§]Geometric Mean and SD for log transformed variable presented

[1]N's 815,953; 557,348; 167,646; 119,650

[2]N's 814,748; 557,466; 167,544; 119,818
