# Supplementary material for: Comorbidity and thirty-day hospital readmission odds in chronic obstructive pulmonary disease: a comparison of the Charlson and Elixhauser comorbidity indices
Source: BMC Health Serv Res. 2019 Oct 15;19:701. doi: 10.1186/s12913-019-4549-4 (PMC6794890; doi:10.1186/s12913-019-4549-4)
Supplement: Supplementary file 12 — Additional file 12: Table S9. Pooled cohort characteristics of patients, stratified by urban/rural hospital location designation. [file 12913_2019_4549_MOESM12_ESM.pdf]

Supplemental Table: Pooled cohort characteristics of patients, stratified by urban/rural hospital location designation.

	Not Readmitted		Readmitted		*P*
	Urban N=1,174,608	Non-Urban N=200,491	Urban N=249,881	Non-Urban N=38,003	
**Sex**, *%*					
Male	40.6%	41.5%	42.5%	43.9%	<.001
Female	59.4%	58.5%	57.5%	56.1%	
**Age**, *Mean ± SD*	67.9 ± 12.3	67.6 ± 10.2	68.7 ± 12.1	68.6 ± 9.9	<.001
**Median household income by ZIP code**, *%*					
1st Quartile	31.4%	61.2%	32.3%	62.5%	<.001
2nd Quartile	26.3%	28.7%	26.0%	28.3%	
3rd Quartile	24.2%	7.1%	23.7%	6.4%	
4th Quartile	16.9%	0.7%	16.9%	0.6%	
Missing	1.2%	2.3%	1.2%	2.3%	
[1]**Patient geographic location**, *%*					
Central county metro area ≥1M	27.1%	0.1%	28.4%	0.1%	<.001
Fringe county metro area ≥1M	29.8%	1.2%	30.6%	1.3%	
County metro area 250,000-999,999k	25.4%	1.7%	24.3%	1.7%	
County metro area 50,000-249,999k	12.4%	1.6%	11.9%	1.5%	
Micropolitan area	2.2%	60.2%	2.1%	60.7%	
Non-metro/non-micropolitan (rural)	3.1%	35.2%	2.8%	34.6%	
[2]**Primary Payer**, *%*					
Medicare (includes dual-eligible)	69.2%	71.2%	74.0%	75.8%	<.001
Medicaid	12.1%	10.3%	13.4%	11.1%	
Private insurance	12.4%	11.6%	8.4%	8.2%	
Self-pay	3.3%	3.5%	1.9%	1.8%	
Other, including no-charge	3.0%	3.4%	2.3%	3.1%	
**Number of admissions each patient had over a year**, *Mean ± SD*	2.14 ± 1.65	2.08 ± 1.35	4.34 ± 2.60	4.12 ± 2.00	<.001
**Number hospitals where each patient received care over a year**, *Mean ± SD*	1.31 ± 0.66	1.31 ± 0.57	1.44 ± 0.77	1.44 ± 0.66	<.001
**Discharge disposition**, *%*					
Routine to home	68.2%	72.8%	59.2%	64.3%	<.001
Transfer to post-acute care	12.7%	11.3%	16.7%	14.1%	
Home with home health services	0.6%	1.2%	0.7%	1.3%	
Other	18.5%	14.7%	23.4%	20.3%	
§**Length of Stay**, *Mean ± SD*	3.73 ± 2.09	3.40 ± 1.45	4.24 ± 2.53	3.77 ± 1.70	<.001
**Care intensity and complications**, *%*					
Use of non-invasive ventilation	8.2%	5.6%	10.2%	7.2%	<.001
Use of mechanical ventilation	4.9%	3.1%	6.0%	4.0%	<.001
Placement or presents of tracheostomy	0.9%	0.4%	1.3%	0.7%	<.001
Cardiac arrest	0.2%	0.1%	0.3%	0.1%	<.001
Performance of CPR	0.1%	0.1%	0.2%	0.1%	<.001

Note: Unweighted N's displayed. Frequencies derived using weighted analysis.
§Geometric Mean and SD for log transformed variable presented
[1]N's 1,172,883; 200,418; 249,306; 37,990
[2]N's 1,172,701; 199,513; 249,519; 37,843
